# Optimal Hormone Replacement Therapy in Hypothyroidism - A Model Predictive Control Approach

**DOI:** 10.3389/fendo.2022.884018

**Published:** 2022-06-24

**Authors:** Tobias M. Wolff, Johannes W. Dietrich, Matthias A. Müller

**Affiliations:** ^1^ Institute of Automatic Control, Leibniz University Hannover, Hannover, Germany; ^2^ Diabetes, Endocrinology and Metabolism Section, Department of Internal Medicine I, St. Josef Hospital, Ruhr University Bochum, Bochum, Germany; ^3^ Diabetes Centre Bochum-Hattingen, St. Elisabeth-Hospital Blankenstein, Hattingen, Germany; ^4^ Ruhr Center for Rare Diseases (CeSER), Ruhr University of Bochum and Witten/Herdecke University, Bochum, Germany

**Keywords:** mathematical modeling, automatic control, model predictive control, thyroid homeostasis, combined therapy, monotherapy

## Abstract

In this paper, we address the problem of optimal thyroid hormone replacement strategy development for hypothyroid patients. This is challenging for the following reasons. First, it is difficult to determine the correct dosage leading to normalized serum thyroid hormone concentrations of a patient. Second, it remains unclear whether a levothyroxine *L*-*T*_4_) monotherapy or a liothyronine/levothyroxine (*L*-*T*_3_/*L*-*T*_4_) combined therapy is more suitable to treat hypothyroidism. Third, the optimal intake frequency of *L*-*T*_3_/*L*-*T*_4_ is unclear. We address these issues by extending a mathematical model of the pituitary-thyroid feedback loop to be able to consider an oral intake of *L*-*T*_3_/*L*-*T*_4_. A model predictive controller (MPC) is employed to determine optimal dosages with respect to the thyroid hormone concentrations for each type of therapy. The results indicate that the *L*-*T*_3_/*L*-*T*_4_ combined therapy is slightly better (in terms of the achieved hormone concentrations) to treat hypothyroidism than the *L*-*T*_4_ monotherapy. In case of a specific genetic variant, namely genotype CC in polymorphism rs2235544 of gene *DIO1*, the simulation results suggest that the *L*-*T*_4_ monotherapy is better to treat hypothyroidism. In turn, when genotype AA is considered, the *L*-*T*_3_/*L*-*T*_4_ combined therapy is better to treat hypothyroidism. Furthermore, when genotype CC of polymorphism rs225014 (also referred to as c.274A>G or p.Thr92Ala) in the *DIO2* gene is considered, the outcome of the *L*-*T*_3_/*L*-*T*_4_ combined therapy is better in terms of the steady-state hormone concentrations (for a triiodothyronine setpoint at the upper limit of the reference range of healthy individuals). Finally, the results suggest that two daily intakes of *L*-*T*_3_ could be the best trade-off between stable hormone concentrations and inconveniences for the patient.

## 1 Introduction

The standard form of therapy to treat hypothyroid patients is a levothyroxine (*L*-*T*_4_) monotherapy (1). *L*-*T*_4_ is a synthetic replacement hormone of thyroxine (*T*_4_) (2). The conversion of *T*_4_ into triiodothyronine (*T*_3_) in peripheral organs like the liver and the kidney as well as the long half-life of *T*_4_ lead to stable serum thyroid hormone concentrations (3, 4).

However, several problems regarding this treatment strategy occur. First, it is difficult to determine the correct individual dosage in practice. Usually, every 4-6 weeks adaptations of the dosages are necessary to reach the desired thyroid stimulating hormone *(TSH)* concentration (1). This results in a long time until the correct individual *L*-*T*_4_ dosage is found and potentially in temporary iatrogen hyperthyroidism (5).

Second, 5 % - 10 % of the hypothyroid patients treated with an *L*-*T*_4_ monotherapy continue suffering from symptoms of hypothyroidism even if their *TSH* concentrations are within the reference range of healthy individuals (6). In order to tackle this second problem, an additional prescription of liothyronine (*L*-*T*_3_) can be considered, leading to a so called *L*-*T*_3_/*L*-*T*_4_ combined therapy. On the one hand, some studies documented that an *L*-*T*_3_/*L*-*T*_4_ combined therapy leads to a higher quality of life and lower depressivity  (7, 8). Additionally, the patients’ own preference tends towards the *L*-*T*_3_/*L*-*T*_4_ combined therapy (7–9). On the other hand, there are studies that do not conclude that the *L*-*T*_3_/*L*-*T*_4_ combined therapy is superior compared to the *L*-*T*_4_ monotherapy regarding aspects as quality of life or symptoms (10–12). A known disadvantage of the *L*-*T*_3_/*L*-*T*_4_ combined therapy is that it usually goes along with undesired fluctuations in the *T*_3_ concentrations (13). By splitting up the daily dosages, these fluctuations can be reduced. However, the best trade-off between stable *T*_3_ concentrations and small inconveniences for the patients is difficult to find.

These issues demonstrate that the existing treatment strategies of hypothyroidism are far from being optimal. One appealing alternative to tackle these issues is to consider a mathematical model of the pituitary-thyroid feedback loop and use it for model-based treatment design. In the pioneering work (14), the authors present a first procedure to determine optimal thyroid hormone replacement strategies based on the solution of one single optimal control problem. However, the applied mathematical model does not take into account a *T*_3_ synthesis which directly depends on the *TSH* concentration (here considered by the so-called *TSH*-*T*_3_ shunt, compare (15)). This aspect will be of crucial importance when comparing the *L*-*T*_4_ monotherapy to the *L*-*T*_3_/*L*-*T*_4_ combined therapy. Furthermore, the drug intake model is quantified by rather old data leading to considerably high thyroid hormone replacement dosages to treat hypothyroidism.

In (16), the authors address the question why some clinical studies document an advantage of the *L*-*T*_3_/*L*-*T*_4_ combined therapy compared to the *L*-*T*_4_ monotherapy, whereas other clinical studies do not document an advantage. The authors apply a mathematical model of the pituitary-thyroid feedback loop to estimate the residual thyroid function. Additionally, they propose optimal steady-state dosages for an *L*-*T*_3_/*L*-*T*_4_ combined therapy in dependence of the residual thyroid function. They formulate and partially validate two hypotheses to explain the different outcomes of the clinical studies. Loosely speaking, the first hypothesis states that patients benefit from the *L*-*T*_3_/*L*-*T*_4_ combined therapy, if their *L*-*T*_3_ dosages bring the *T*_3_ concentrations to the upper reference range of healthy individuals, whereas their *L*-*T*_4_ dosages normalize the *T*_4_ concentrations. The second hypothesis states that the residual thyroid function substantially influences the outcome of an *L*-*T*_3_/*L*-*T*_4_ combined therapy. However, compared to our work, no controller is developed to determine optimal dosages and no genetic variants are considered.

The authors in (17) propose a simplified mathematical model of the pituitary-thyroid feedback loop and illustrate the influence of different thyroid hormone replacement dosages on the hormone concentrations in the case of hypothyroidism. However, the applied model of that work does not consider the *T*_3_ concentrations. Consequently, no comparison between an *L*-*T*_4_ monotherapy and an *L*-*T*_3_/*L*-*T*_4_ combined therapy is possible. Furthermore, no oral intake of the thyroid hormone replacement dosages is considered but rather an “intravenous intake”. This procedure prevents an analysis of the intake frequency of the medication dosages on the thyroid hormone concentrations.

Recently, a clinical trial evaluated the treatment outcome of thyroidectomized patients when the responsible physician is supported by a decision aid tool (18). This tool is based on an individualized *TSH*-*T*_4_ relationship and a simple dynamic model for the course of free *T*_4_ (*FT*_4_). An optimal *L*-*T*_4_ steady-state dosage is determined by means of mathematical optimization and proposed to the treating physician. Again, the *T*_3_ concentrations (and consequently an *L*-*T*_3_ prescription) are not considered in the mathematical model and, additionally, the authors assume an “intravenous intake” of *L*-*T*_4_ for the computations of the optimal dosages.

In this work, we extend the mathematical model of the pituitary-thyroid feedback loop originally developed by (19) to consider the oral drug intake of *L*-*T*_3_ and *L*-*T*_4_. We then design a model predictive controller (MPC) for the pituitary-thyroid feedback loop to determine optimal thyroid hormone replacement strategies for both types of therapy, different genetic variants and different frequencies of drug intake.

This procedure leads to several contributions of this paper. First, the approach paves the way to improve the current trial-and-error process of prescribing thyroid replacement hormones, since the optimal dosages need not to be estimated by a physician, but can directly be determined by the controller. Second, simulations of both treatment strategies show that the *L*-*T*_3_/*L*-*T*_4_ combined therapy is slightly superior to treat hypothyroidism compared to the *L*-*T*_4_ monotherapy both in case without genetic variants and for genotype AA of polymorphism rs2235544 of gene *DIO1*. However, given genotype CC of polymorphism rs2235544, an *L*-*T*_4_ monotherapy can turn out to be advantageous compared to an *L*-*T*_3_/*L*-*T*_4_ combined therapy. An *L*-*T*_3_/*L*-*T*_4_ combined therapy can be beneficial in terms of the steady-state hormone concentrations for patients with the CC genotype of polymorphism rs225014 (also referred to as c.274A>G or p.Thr92Ala) of gene *DIO2* for a *T*_3_ setpoint at the upper limit of the reference range of healthy individuals. Third, we analyze the impact of one, two, or three daily intakes of *L*-*T*_3._ Two daily intakes could be the best trade-off between stable *T*_3_ concentrations and a convenient therapy for the patient.

## 2 Methods

The basis of this work is a mathematical model of the pituitary-thyroid feedback loop. Loosely speaking, the (simplified) operating principle of this feedback loop is the following: the production of the thyroid hormones *T*_3_ and *T*_4_ is stimulated by *TSH*. Additionally, *T*_4_ is converted into *T*_3_ in peripheral organs and in the thyroid by means of 5’-deiodinase type I (D1) and 5’-deiodinase type II (D2). The production of *TSH* is inhibited by thyroid hormones and stimulated by thyrotropin-releasing hormone (*TRH*).

**Figure 1:**
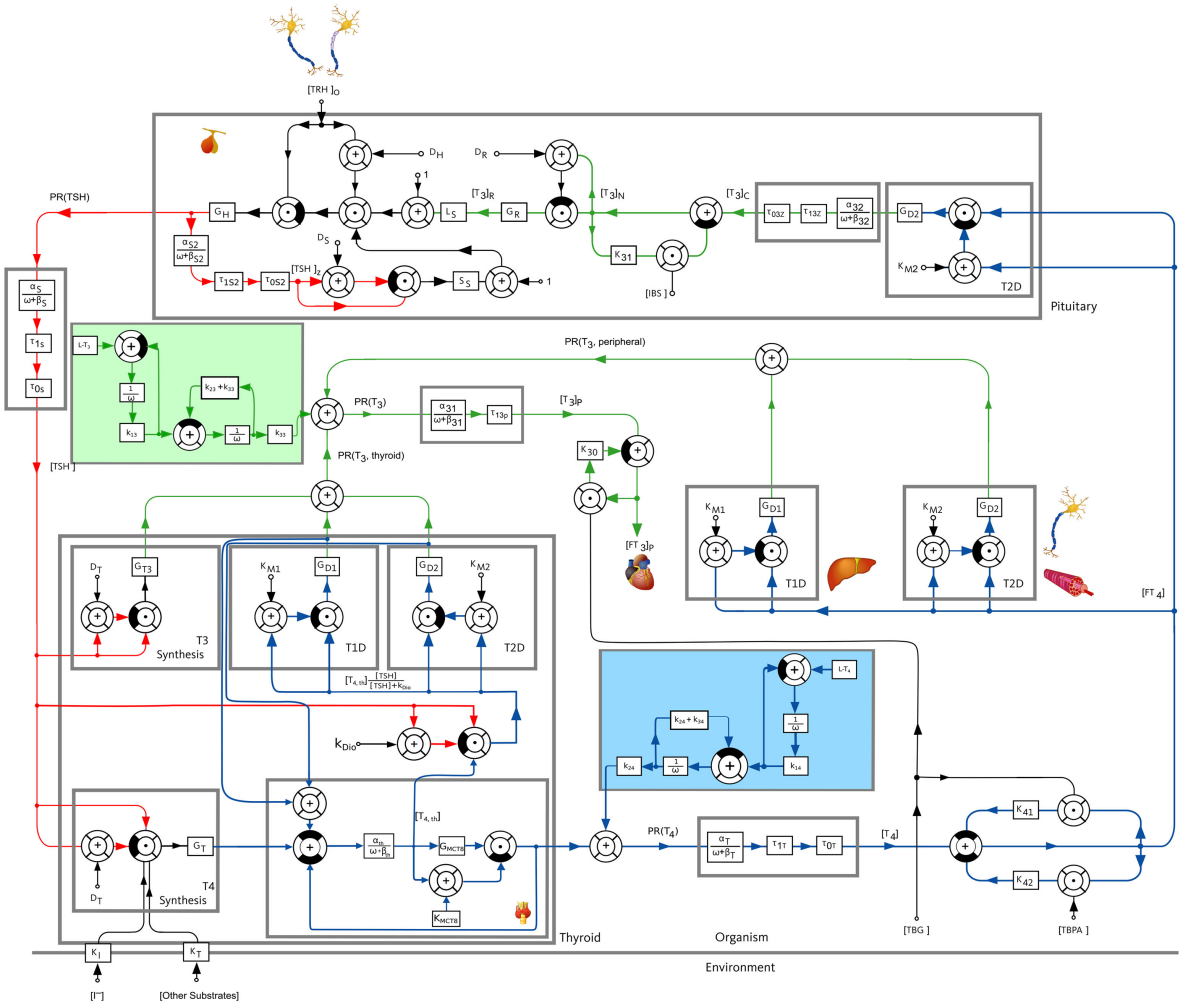
Block diagram of the pituitary-thyroid feedback loop. A detailed explanation of the model is given in (20, **Supplementary Material**). The underlying differential equations are shown in **Section S1 of the Supplementary Material** and the numerical parameter values are given in **Section S4 of the Supplementary Material**. The extensions of the mathematical model compared to the previous versions of the model, see (15, 19, 20), are shown by a slight blue and a slight green background color.

In this work, we consider a mathematical model of the pituitary-thyroid feedback loop which was originally developed in (19) and extended in (15, 20). It consists of six nonlinear differential equations describing the cause-effect relations between different hormone concentrations, compare **Section S1 of the Supplementary Material** for the exact definition of the differential equations. A more detailed explanation of this model and the underlying mechanisms can be found in (20, **Supplementary Material**). The complete mathematical model is illustrated in **Figure 1**.

The first step of our work is to extend the mathematical model once again to be able to consider the oral medication intake of *L*-*T*_3_ and *L*-*T*_4_. To this end, we adopt the model used in (14), a two compartment model describing the intake, dissolution and absorption of thyroid replacement hormones. By means of a least-squares fit, we fit the numerical values of the constants *k*_1_ *_i_* (dissolution rate constant), *k*_2_ *_i_* (direct gut-excretion rate constant) and *k*_3_ *_i_* (absorption rate constant) to the more recent results documented in (21), rather than to the ones used by (14), namely (4). In (21), the authors document a dependence of the bioavailabilities of *L*-*T*_3_ and *L*-*T*_4_ on the thyroid state of the patient (euthyroid, hypothyroid, hyperthyroid). Throughout this work, we apply the bioavailability of euthyroid individuals. In addition, we perform simulations using the bioavailability of hypothyroid patients which demonstrate that the results remain qualitatively the same for both bioavailabilities (compare **Section S3.3 of the Supplementary Material**). Nevertheless, an interesting topic for further research would be to dynamically adapt the bioavailabilities in dependence of the thyroid state. The extensions of the model are shown in **Figure 1** with a slight green and blue background color regarding the intake of *L*-*T*_3_ and *L*-*T*_4_, respectively. The exact numerical parameter values of the oral medication intake model as well as of the complete mathematical model are shown in **Section S4 of the Supplementary Material**.

In its original form, the mathematical model represents generic euthyroid healthy individuals, since the parameters of the model are either well-known quantities (such as the half-life of thyroid hormones) or fitted to serum thyroid function tests of healthy individuals see (20). Hypothyroidism can be considered in the mathematical model by choosing a smaller value for the secretory capacity of the thyroid gland (*G_T_*) (15). In this work, we choose a secretory capacity of the thyroid, which represents 10 % of the one of healthy individuals. Note that the secretory capacity of thyroidectomized hypothyroid patients would correspond to *G_T_* = 0. Furthermore, the parameter *G_T_* could also be determined individually by means of the method SPINA- *G_T_* (22). Therefore, extending the results presented here for generic hypothyroid patients to individual patients by fitting *G_T_* and other model parameters as *G_D_*_1_ (maximum activity of D1) and *G_T_*_3_ (maximum activity of direct *T*_3_ synthesis) to individual patient data is an interesting topic for future research; we emphasize that the main findings of the paper are qualitatively the same for other values of *G_T_* (and *G_D_*_1,_ *G_T_*_3_) compare **Section S3.1 of the Supplementary Material** for further simulation results considering different numerical values of *G_T_* than the one used in the main part. For the sake of brevity, the simulation results regarding different *G_D_*_1_/*G_T_*_3_ parameter configurations are not shown here.

Next, we focus on how optimal dosages of the *L*-*T*_4_ monotherapy and the *L*-*T*_3_/*L*-*T*_4_ combined therapy can be determined. To this end, we make use of MPC, which is one of the most successful modern control methods (23). The potentially most important advantage is that constraints can be incorporated in the design of the controller, which is typically not the case for other control methods. Loosely speaking, the principle of MPC is the following: at each sampling instant, the optimal input trajectory with respect to a cost function is determined by predicting the system’s behavior over a specific time into the future. Only the first element of the optimal input trajectory is then applied to the system and the system’s state is measured again. This procedure is repeated until a predefined number of iterations is elapsed. In our case, the cost function denotes a weighted quadratic difference between the hormone concentrations *T*_3_, *T*_4_, and *TSH* and their equilibrium hormone concentrations of generic healthy individuals^^1^^ (the setpoint to be reached via medication intake), compare **Section S2 of the Supplementary Material** for a detailed mathematical description of the MPC setting and further comments on the design of MPC.

As mentioned in the previous section, we want to analyze the effect of specific genetic variants on the outcome of both types of therapy. In this work, we focus exemplarily on polymorphism rs2235544 in gene *DIO1* encoding D1, which is associated with different D1 activities in dependence of the number of C-allele (24). To model such genotypes, we exemplarily reduce and increase the maximal activity of D1 (named *G_D_*_1_ that was determined by means of real thyroid hormone measurements of healthy individuals, compare footnote 1) in the mathematical model by 10 %. In other words, the genotype AA of polymorphism rs2235544 is modeled by reducing *G_D_*_1_ to *G*_*D*1_'=0.9*G*_*D*1_ and the genotype CC of polymorphism rs2235544 is modeled by increasing *G_D_*_1_ to *G*_*D*1_''=1.1*G*_*D*1_. In addition, we consider the case in which the CC genotype is modeled by *G*_*D* 1_'''=1.2*G*_*D* 1_, which yields additional insight regarding the evaluation of the therapies. We determine optimal thyroid hormone replacement strategies based on an *L*-*T*_4_ monotherapy and an *L*-*T*_3_/*L*-*T*_4_ combined therapy for these three cases. In addition, we consider the genotype CC of polymorphism rs225014 that is associated with a decreased D2 activity (25) and with an improved response (in terms of symptoms) to the *L*-*T*_3_/*L*-*T*_4_ combined therapy (26). To model this variant, we reduce exemplarily *G_D_*_2_ to *G*_*D*2_'=0.6*G*_*D*2_, corresponding to the results documented in (25).

Finally, we analyze the optimal frequency of medication intake. In general, a higher frequency of medication intake, e.g., two daily drug intakes instead of one daily drug intake result in more stable hormone concentrations. This is advantageous since variations of the thyroid hormone concentrations lead to an increased cardiovascular risk (27). However, a higher frequency of medication intake is less convenient for the patient. Therefore, we consider the cases when the daily dosage is taken once, twice, or three times per day. A good medication outcome should result in hormone concentrations that are as monotone as possible and not fluctuating too much. Regarding the transient phase, it means that the hormone concentrations shall rise as smoothly as possible, until the desired euthyroid setpoint is reached. After reaching the desired euthyroid setpoint, the hormone concentrations shall stay as constant as possible. Both effects can be captured by calculating how much the hormone concentrations decrease within one day, compare **Table 1** in Section 4.1 for the exact formula.

**Table 1:** Effects of the frequency of medication intake on the fluctuations of the *T*_3_ and the *T*_4_ concentrations for the first and the 15th day of therapy regarding the *L*-*T*_4_ monotherapy and the *L*-*T*_3_/*L*-*T*_4_ combined therapy.

	One Daily Intake		Two Daily Intakes		Three Daily Intakes	
Day of therapy	1	15	1	15	1	15
	***L*-*T*_4_ monotherapy**					
Δ*T*_3_	0%	0.6%	0%	0.2%	0%	0.1%
Δ*T*_4_	6.5%	8.6%	3.1%	4.0%	1.9%	2.6%
	***L*-*T*_3_/*L*-*T*_4_ combined therapy**					
Δ*T*_3_	13.2%	5.2%	5.8%	2.6%	3.4%	1.6%
Δ*T*_4_	6.5%	8.4%	3.1%	4.0%	1.9%	2.6%

The abbreviations ΔT_3_ /ΔT_4_ describe the term $\frac{Max-Min}{Min}$ of the T_3_ and T_4_ concentrations, respectively. The Min value is the minimal hormone concentration after the daily peak has been reached. The Max value is the maximal daily hormone concentration. This means that we quantify the daily decrease of the hormone concentrations. If the hormone concentrations monotonically increase throughout the complete day, no fluctuations in the sense of the applied definition are present.

Throughout this work, we consider a constant *TRH* concentration, compare **Section S4 of the Supplementary Material** for the exact numerical value. Obviously, a constant *TRH* concentration is an approximation of the real pulsatile *TRH* course. This approximation can be used in this work because mainly *TSH* is affected by the course of *TRH* (15). Here, we are interested in the concentrations of *T*_3_ and *T*_4_, which are only slightly affected by the pulsatile *TRH* course (15).

## 3 Results

First, we focus on the simulations of the *L*-*T*_4_ monotherapy and the *L*-*T*_3_/*L*-*T*_4_ combined therapy for hypothyroid patients^^2^^ for one daily drug intake. Second, the simulated treatment of different genetic variants is shown. Third, the results regarding the impact of the frequency of medication intake are presented. Note that the hormone concentrations are in their (hypothyroid) steady state prior to the beginning of the thyroid hormone replacement therapies.

The results were obtained by means of a standard PC (Intel(R) Core(TM) i7-10875H CPU @ 2.30GHz (16 CPUs) processor with 16 GB RAM) using MATLAB/Simulink^®^, version 9.10.0.1684407 (R2021a), CasADi (28) and the NLP solver IPOPT (29).

### 3.1 L-T_4_ Monotherapy

**Figure 2:**
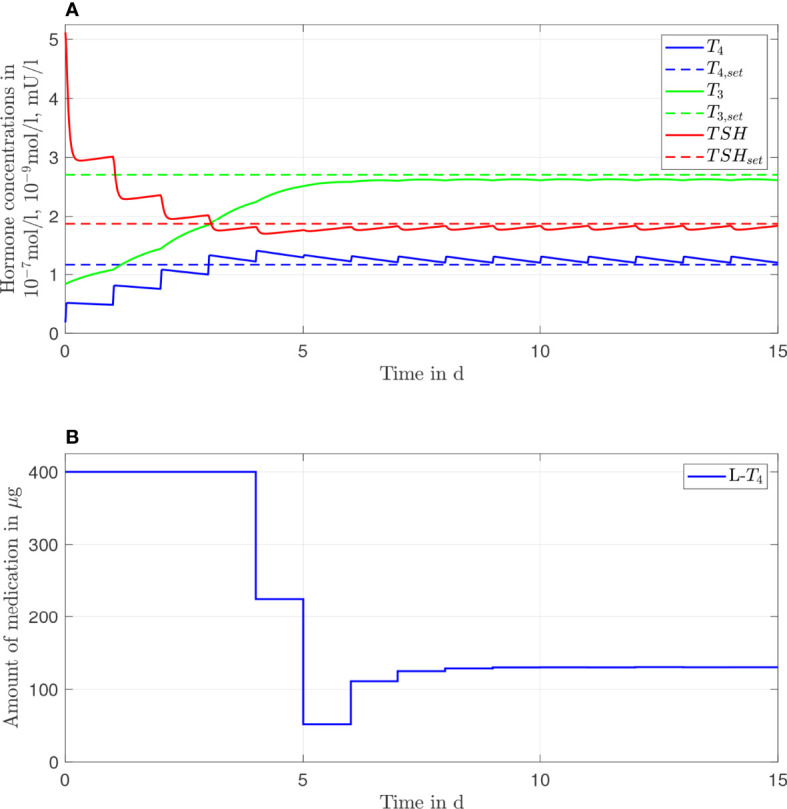
Simulation results of an L-T_4_ monotherapy for a generic hypothyroid patient. The hormone concentrations **(A)** result from the application of the thyroid hormone replacement dosages **(B)**, which are determined by means of an MPC.

In **Figure 2**, the results of the *L*-*T*_4_ monotherapy are illustrated for one daily intake, meaning that the simulated treatment considers one daily intake of *L*-*T*_4_. In **Figure 2A**, the course of the hormone concentrations over 15 days is illustrated by the continuous lines. The dashed lines represent the setpoints of the considered euthyroid generic healthy individual. **Figure 2B** illustrates the corresponding amount of *L*-*T*_4_ for each day. The hormone concentrations do not reach their desired setpoints. The concentration of *T*_4_ remains slightly higher, and the concentrations of *TSH* and *T*_3_ remain slightly lower than their respective setpoints. The concentrations of *T*_4_ and *TSH* fluctuate strongly compared to the concentration of *T*_3_. A high starting dosage (400 µg *L-T*_4_) followed by a steady-state dosage (130 µg) are optimal. The nonlinear decrease of the *TSH* concentrations is due to the nonlinear dependence of *T*_4_ and *TSH*, compare eqs. (S4), (S5), (S8), and (S9) in the **Supplementary Material**.

### 3.2 L-T_3_/L-T_4_ Combined Therapy

**Figure 3:**
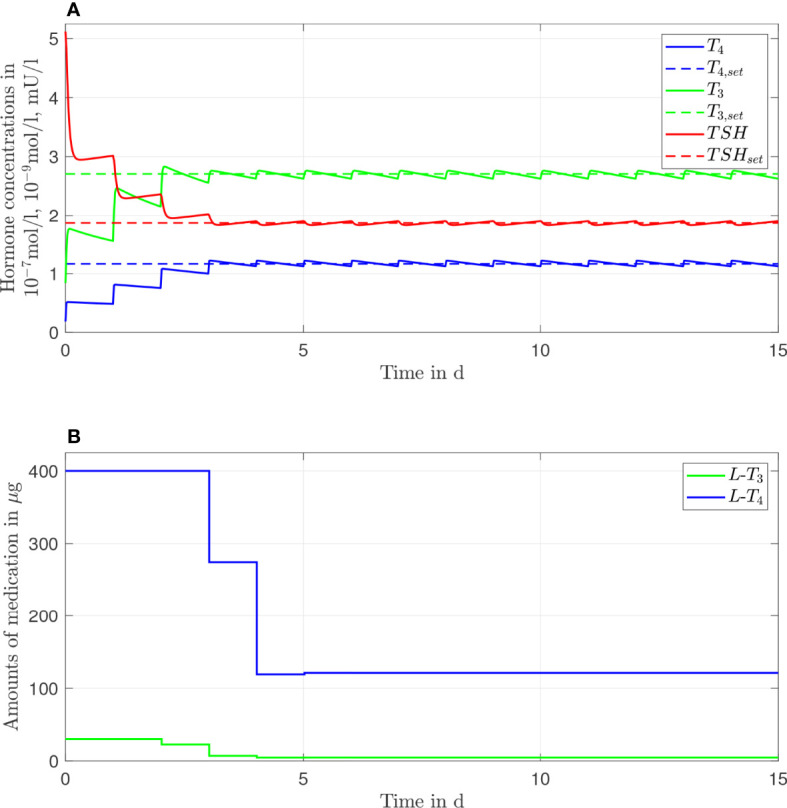
Simulation results of an L-T_3_/L-T_4_ combined therapy for a generic hypothyroid patient. The hormone concentrations **(A)** result from the application of the thyroid hormone replacement dosages **(B)**, which are determined by means of an MPC.

In **Figure 3**, the simulation results of the *L*-*T*_3_/*L*-*T*_4_ combined therapy are illustrated for one daily intake. In contrast to the *L*-*T*_4_ monotherapy, the setpoint is reached for all hormone concentrations (taking into account the unavoidable daily fluctuations), no persisting offset between the hormone concentrations and their setpoints can be seen. The fluctuations of the hormone concentration of *T*_3_ are higher, whereas the fluctuations of *T*_4_ and *TSH* are similar compared to the *L*-*T*_4_ monotherapy. Again, high starting dosages (400 µg of *L*-*T*_4_ and 30 µg of *L*-*T*_3_) followed by steady-state dosages (121 µg of *L*-*T*_4_ and 4.7 µg of *L*-*T*_3_) are optimal.

### 3.3 Genetic Variants

**Figure 4:**
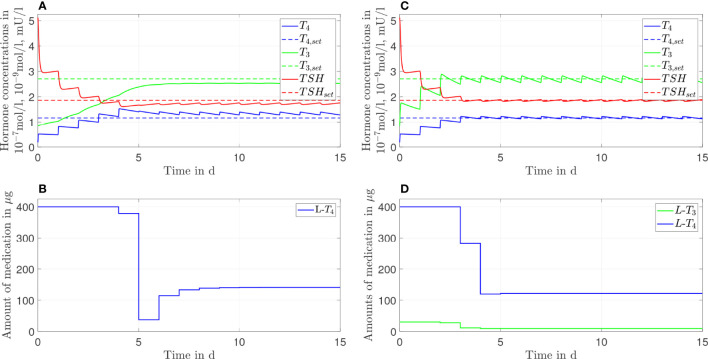
Simulation results of an *L-T_4_* monotherapy **(A, B)** and an *L-T_3_*/*L-T_4_* combined therapy **(C, D)** for G'_D1_ = 0.9G_D1_.

**Figure 5:**
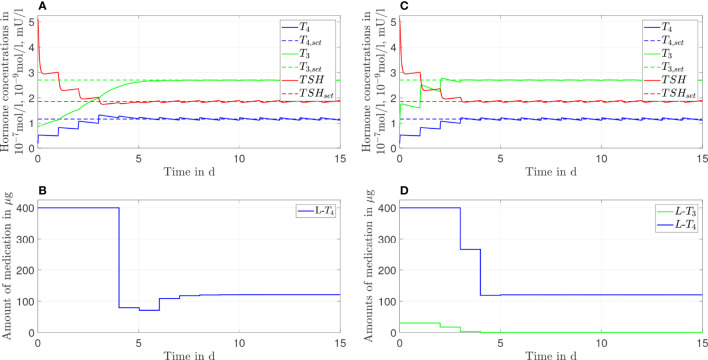
Simulation results of an *L-T_4_* monotherapy **(A, B)** and an *L-T_3_/L-T_4_* combined therapy **(C, D)** for G''_D1_ = 1.1G_D1_.

**Figure 6:**
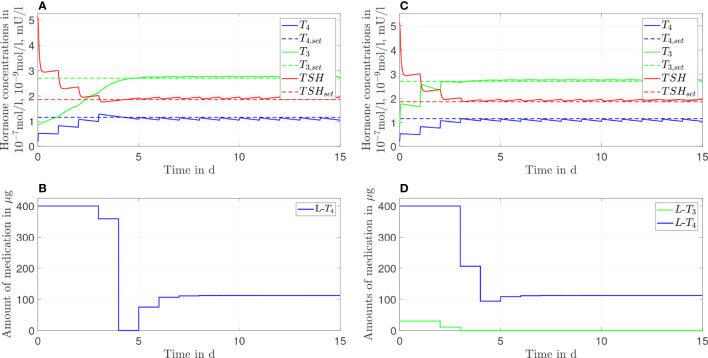
Simulation results of an *L-T_4_* monotherapy **(A, B)** and an *L-T_3_/L-T_4_* combined therapy **(C, D)** for G'''_D1_ = 1.2G_D1_.

In this section, the results of both therapies for different genetic variants are shown. In **Figures 4**–**6**, we consider *G*_*D*1_'=0.9*G*_*D*1_, *G*_*D*1_''=1.1*G*_*D*1_, *G*_*D* 1_'''=1.2*G*_*D* 1_, respectively. The left column plots illustrate the results of the *L*-*T*_4_ monotherapy and the right column plots show the results of the *L*-*T*_3_/*L*-*T*_4_ combined therapy. As mentioned in the previous section, the adapted numerical values represent exemplarily different genotypes.

The *L*-*T*_4_ monotherapy does not restore the setpoint of healthy individuals for the case *G*_*D*1_'=0.9*G*_*D*1_ (compare **Figures 4A, B**). There is a substantial offset visible between the *T*_3,_ *T*_4,_ and *TSH* concentrations and their euthyroid setpoints. This offset is higher compared to the case when no genetic variant is considered, compare **Figure 2**. In turn, the *L*-*T*_3_/*L*-*T*_4_ combined therapy restores the euthyroid setpoint of healthy individuals up to some daily fluctuations.

In the case of *G*_*D*1_''=1.1*G*_*D*1_ (compare **Figure 5**), not only the *L*-*T*_3_/*L*-*T*_4_ combined therapy but also the *L*-*T*_4_ monotherapy reaches the euthyroid setpoint. In contrast to the *L*-*T*_4_ monotherapy, the *L*-*T*_3_/*L*-*T*_4_ combined therapy goes along with high fluctuations of *T*_3_ during the first days of therapy.

**Figure 7:**
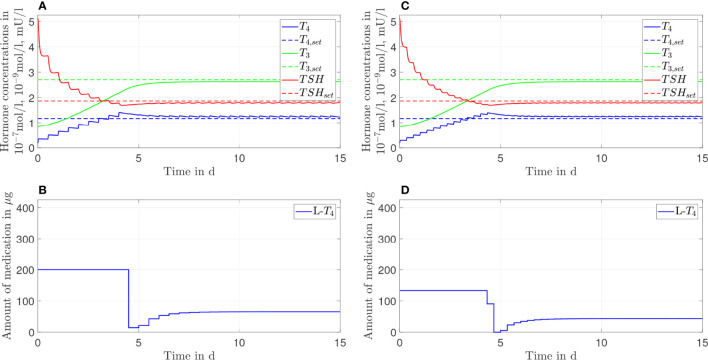
Simulation results of an *L-T_4_* monotherapy in which the patient takes in a drug two times **(A, B)** or three times **(C, D)** a day.

**Figure 8:**
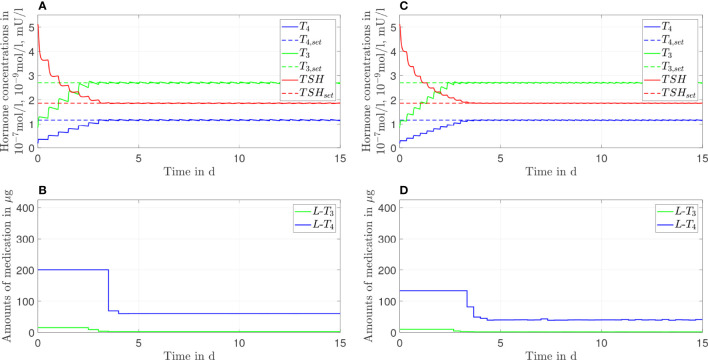
Simulation results of an *L-T_3_/L-T_4_* combined therapy in which the patient takes in a drug two times **(A, B)** or three times **(C, D)** a day.

In the last case, where *G*_*D* 1_'''=1.2*G*_*D* 1_, the *L*-*T*_4_ monotherapy and the *L*-*T*_3_/*L*-*T*_4_ combined therapy lead to similar results. Both therapies reach the setpoint up to some small offset; the concentrations of *T*_3_ and *TSH* are slightly higher than their respective setpoints whereas the concentration of *T*_4_ remains slightly lower than the respective setpoint, which is the opposite situation to **Figure 2**. In **Figure 6D**, one can see that after day three no *L*-*T*_3_ is needed to remain in the steady state. Therefore, the *L*-*T*_3_/*L*-*T*_4_ combined therapy reduces to an *L*-*T*_4_ monotherapy. For the sake of brevity, the results concerning the CC genotype of polymorphism rs225014 are shown in **Figures 7**, **8** of the **Supplementary Material**. These figures illustrate that the *L*-*T*_3_/*L*-*T*_4_ combined therapy results in better steady-state hormone concentrations, if the *T*_3_ setpoint is set to the upper limit of the reference range of healthy individuals. Additionally, the euthyroid setpoint is reached earlier in case of the *L*-*T*_3_/*L*-*T*_4_ combined therapy.

### 3.4 Frequency of Medication Intake

In this section, we show the results of the analysis regarding the frequency of medication intake. Here, we do not consider genetic variants. We compare one, two, and three daily intakes. Since **Figures 2**, **3** already show the results for one daily intake, **Figures 7**, **8** complement the cases for two and three daily intakes. **Table 1** summarizes the effects of each frequency of intake for both types of therapy. Exemplary, we analyze the first day and the last day of therapy, which are representative for the transient phase and the steady state.

In general, concerning both types of therapy, an increasing number of medication intakes reduces the fluctuations of all hormone concentrations. In particular, we focus on the concentrations of *T*_3_. Regarding the *L*-*T*_4_ monotherapy (compare **Figures 2**, **7**), the *T*_3_ concentrations do not fluctuate and smoothly increase until the steady state is reached.

In turn, the *T*_3_ concentrations fluctuate substantially concerning the *L*-*T*_3_/*L*-*T*_4_ combined therapy (see **Figures 3**, **8**). These fluctuations are higher in the transient phase and smaller in the steady state. The strongest fluctuations occur for one daily intake.

Note that the small variations of the *L*-*T*_4_ dosage (after the steady state has been reached) regarding three daily medication intakes (compare **Figure 8D**) are due to numerical issues and not due to medical reasons. We need to solve a highly nonlinear optimization problem where the solver can be stuck in local minima.

## 4 Discussion

### 4.1 Prescription Policy

As mentioned in the introduction, one difficulty of the current treatment strategies is the trial-and-error process in order to find the correct individual dosage. The here presented procedure represents the first step to improve this (potentially unnecessary) trial-and-error process. For a generic hypothyroid patient, the daily dosages can directly be adopted from the simulation results of the MPC. In contrast to (18), the MPC does not only compute an optimal steady-state dosage but also optimal dosages regarding the transient phase of the therapy.

Furthermore, the usage of an MPC provides the opportunity to adapt the dosage much earlier, if necessary. In **Figures 2**, **3**, one can see that the steady-state hormone concentrations are reached within the first week of the start of the therapy. Therefore, if the resulting thyroid hormone replacement strategy does not yield to the desired outcome, one can adapt the dosage(s) already after one or two weeks, as it is also suggested in (18). It is not necessary that the adaptation is made after 4-6 weeks, as it is currently recommended (1).

Another interesting aspect is the tendency that high dosages at the beginning of the therapy followed by lower dosages are optimal. In the past, some experts suggested that the treatment strategy should start with low dosages and should be continued by increasing the dosages only slowly (sometimes designated as “start low go slow” policy), especially for elderly patients (1, 30). However, this strategy seems outdated for cardiac asymptomatic patients (31). The results presented here underline once again the advantageous effects of high starting dosages: the setpoint is reached much faster with this policy than by following the “start low go slow” policy. These considerations apply especially to myxoedema coma, the most severe complication of hypothyroidism. The question of optimal substitution treatment remains controversial (32), and the modality of optimum treatment continues to be uncertain, mainly due to the low number of clinical studies and inherent difficulties in performing controlled trials. The population at risk from myxoedema coma predominantly includes elderly subjects and patients suffering from cardiovascular disease, thereby suggesting that the dosage should be escalated slowly. However, several case series and small studies reported it to be beneficial to start with a single high-dose intravenous bolus of 500 µg and to continue with a maintenance dose of 50 to 100 µg daily (33, 34).

One important long-term perspective of this work is to translate these results into clinical practice. Before this translation can be realized, two issues must be considered. First, the optimal dosages determined by means of the MPC may not be available in form of a commercial product. Second, the medication dosages should not be adapted every day. As visible in **Figure 2B**, the results of the MPC suggest that the (generic) patient should take in 400 µg of *L*-*T*_4_ for the first four days. In the following days, the patient should take in several different dosages before a constant dosage of approximately 130 µg of *L*-*T*_4_ should be taken in. In clinical practice, one might simply prescribe 400 µg of *L*-*T*_4_ for the first four days. Subsequently, one could directly prescribe the closest commercially available dosage to 130 µg of *L*-*T*_4_. Following this procedure, one only adapts the dosage once and additionally only uses dosages that are available in form of a commercial product. An additional issue must be considered regarding the prescription of *L*-*T*_3_. Currently, the commercially available *L*-*T*_3_ dosages are remarkably higher than the dosages that are recommended in clinical guidelines and that are determined in this work. For example, in Germany, the available *L*-*T*_3_ medications such as Thybon^®^ 20 or the combined *L*-*T*_3_/*L*-*T*_4_ preparation Prothyrid^®^ contain 20 µg or 10 \mu\g of L-T_3_, *L*-*T*_3_ respectively. These dosages are approximately four times or two times higher compared to the optimal dosages determined by means of the MPC in this work. Therefore, the implementation of our work (and of the mentioned guidelines) in clinical practice imperatively requires the availability of pharmaceutical preparations containing lower dosages of *L*-*T*_3_.

Most practicing physicians are familiar with the prescription of the dosages regarding an *L*-*T*_4_ monotherapy. However, they are potentially not familiar with the prescription of *L*-*T*_3_ making the treatment with an *L*-*T*_3_/*L*-*T*_4_ combined therapy challenging. Possible guidelines for physicians are given by the European Thyroid Association (13). Four alternative formulas are given, but no preference regarding one formula is mentioned. Consequently, the treating physician decides the formula so far. We provide assistance to this decision by comparing the formulas of (13) to our simulation results. The formulas in (13) are based on a prior treatment with *L*-*T*_4_ that normalizes the thyroid hormone concentrations, which would be in our case around 130 \mu \g, compare **Figure 2**. The resulting dosages for the *L*-*T*_3_/*L*-*T*_4_ combined therapy of the four different formulas are given in **Table 2**. The dosages regarding an *L*-*T*_3_/*L*-*T*_4_ combined therapy determined by means of the MPC are approximately 121 µg of *L*-*T*_4_ and 4.7 of *L*-*T*_3_. By comparing these dosages to the ones given in **Table 2**, one can conclude that the simulation results fit best to formula B2. This suggests that from the four different methods proposed in (13), formula B2 seems to be the best strategy.

**Table 2:** Recommended dosages regarding an *L*-*T*_3_/*L*-*T*_4_ combined therapy based on a prior treatment with an *L*-*T*_4_ monotherapy (here with 130 µg of *L*-*T*_4_) and the four different formulas given in (13).

	Method A	Method B1	Method B2	Method C	MPC
*L*-*T*_3_ in μg	7.6	6.1	5.4	6.5	4.7
Δ*L*-*T*_3_	61.7%	23.0%	14.9%	27.7%	—
*L*-*T*_4_ in μg	107.1	122.4	122.5	110.5	121
Δ*L*-*T*_4_	13.0%	1.1%	1.2%	9.5%	—

The abbreviations ΔL-T_3_ and ΔL-T_4_ stand for $\frac{\left|Method\;i-MPC\right|}{Method\;i}$ for i = {A, B1, B2, C}.

So far, we only considered a generic hypothyroid patient. A matter of ongoing research deals with a reliable individualization of the mathematical model. When this is the case, the treatment strategies obtained by applying the MPC will be optimal individual strategies. This would clearly pave the way into the era of personalized medicine. In a first clinical trial exploiting the advantages of mathematical modeling to determine optimal dosages, the importance of an individualized model is pointed out (18). However, the individualization of our complex model is currently not straight-forward, one problem in this context is that some model parameters cannot be identified uniquely based on only few individual hormone measurements (15, 20). This issue could potentially be resolved using more frequently measured (dynamic) hormone data as available in (18), compare the discussions in (15) and (20), which is subject of future work.

Interestingly, clinical studies such as (35) document that athyroid patients that are treated with an *L*-*T*_4_ monotherapy have higher *T*_4_ but similar *T*_3_ concentrations compared to the patients’ prethyroidectomy concentrations. Higher *T*_4_ concentrations in the case of the *L*-*T*_4_ monotherapy are also visible in the simulations of the mathematical model, compare **Figure 2**. However, the simulations show slightly decreased concentrations of *T*_3_ compared to the concentrations of healthy individuals, see **Figure 2**. This can be explained by the design of the MPC (compare **Section S2 of the Supplementary Material**). The objective is to reach simultaneously the euthyroid steady-state concentrations of *T*_3_, *T*_4_ and *TSH* leading to a trade-off between too high *T*_4_ and too low *T*_3_ concentrations. If one chooses a cost function that penalizes solely the deviation of *T*_3_ from its steady state, the result is a *T*_3_ conncentration similar to the setpoint of healthy individuals and a higher concentration of *T*_4_ than the one observed in **Figure 2** as reported in (35). For the sake of brevity, the corresponding simulation results are illustrated in **Section S3.2, ****Figure 3**** of the Supplementary Material**. Additionally, that section contains simulation results with cost functions that penalize mainly the deviations of *T*_4_ or *TSH* from their euthyroid setpoints. These results illustrate that the course of the hormone concentrations depends on the tuning of the weighting matrix *Q*, compare **Figures 4** and **5** of the **Supplementary Material**.

Note that the authors in (16) underline the importance of high serum *T*_3_ concentrations for a successful outcome of the *L*-*T*_3_/*L*-*T*_4_ combined therapy. This objective could be easily met by adapting the cost function of the MPC so that the difference between the measured hormone concentrations and the upper limit of the reference range is penalized. In addition, for patients affected by differentiated thyroid cancer a cost function that solely penalizes the *T*_3_ concentrations can be benefical, since for these patients the free T_3_ (*FT*_3_) concentratios are particulary important for the relief of symptoms, compare (36).

### 4.2 Comparison L-T_4_ Monotherapy With L-T_3_/L-T_4_ Combined Therapy

As mentioned in the previous section, the *L*-*T*_4_ monotherapy can only approximately restore the setpoint of healthy individuals (with some remaining offset), whereas the *L*-*T*_3_/*L*-*T*_4_ combined therapy can restore this setpoint. In the case of the *L*-*T*_4_ monotherapy, the missing endogenously produced *T*_4_ is simply replaced by *L*-*T*_4_. *T*_3_ is synthesized out of *T*_4_ in peripheral organs and in the thyroid mainly by D1 and by D2. The peripheral *T*_3_ production is restored by an *L*-*T*_4_ monotherapy, since the serum *T*_4_ is replaced by *L*-*T*_4_. In turn, the production of *T*_3_ in thyroid cells (describing the *TSH*-*T*_3_ shunt) cannot be restored by *L*-*T*_4_, because *L*-*T*_4_ only enters the pituitary-thyroid feedback loop in the periphery and not within the thyroid. Consequently, the *L*-*T*_4_ dosages increase, until the best trade-off between a too low concentration of *T*_3_ and a too high concentration of *T*_4_ is found.

Regarding the *L*-*T*_3_/*L*-*T*_4_ combined therapy, the missing endogenously produced *T*_4_ and the subsequent peripheral *T*_3_ production can again be replaced by *L*-*T*_4_. In contrast to the *L*-*T*_4_ monotherapy, the production of *T*_3_ in thyroid cells is compensated through the intake of *L*-*T*_3_. Since only a small fraction of *T*_3_ is synthesized within thyroid cells (15), the necessary *L*-*T*_3_ dosages are rather low.

As explained above, the main reason for the resulting different treatment outcome of an *L*-*T*_4_ monotherapy compared to an *L*-*T*_3_/*L*-*T*_4_ combined therapy is the fact that the *T*_3_ production also takes place in thyroid cells (the so-called *TSH*-*T*_3_ shunt, compare (15)). When this intrathyroidal *T*_3_ production is not considered in the mathematical model, the final treatment outcome between the two types of therapy is the same. In particular, in (14), where no direct *T*_3_ synthesis based on *TSH* is considered in the model (also referred to as *TSH*-*T*_3_ shunt), the authors conclude that the *L*-*T*_3_/*L*-*T*_4_ combined therapy is not superior to the *L*-*T*_4_ monotherapy, because the same hormone concentrations are reached and because of undesired fluctuations of the *T*_3_ concentrations in the case of the *L*-*T*_3_/*L*-*T*_4_ combined therapy.

Since the difference in the outcome of the two therapies is rather small (compare **Figures 2**, **3**), it might seem questionable whether an *L*-*T*_3_/*L*-*T*_4_ combined therapy should be recommended in practice. However, when it is known that the patient is affected by an AA or CC genotype in polymorphism rs2235544 in *DIO1*, the difference between the *L*-*T*_4_ monotherapy and the *L*-*T*_3_/*L*-*T*_4_ combined therapy in the simulations of the model can be substantial. On the one hand, when patients have an AA genotype (leading to lower activity of D1, here exemplary considered by *G*_*D*1_'=0.9*G*_*D*1_) they might profit considerably from an *L*-*T*_3_/*L*-*T*_4_ combined therapy, compare **Figure 4**. In this case, less *T*_3_ is produced out of *T*_4_ in peripheral organs and in the thyroid gland. The lack of endogenously produced *T*_3_ cannot be compensated by means of the *L*-*T*_4_ monotherapy. Again, the controller increases the *L*-*T*_4_ dosages further in order to reach the best trade-off between a too low *T*_3_ concentration and a too high *T*_4_ concentration. In turn, the *L*-*T*_3_/*L*-*T*_4_ combined therapy restores the setpoint of healthy individuals by means of higher *L*-*T*_3_ dosages. The difference to the case without genetic variants is an increased offset between the *T*_3_, *T*_4_ and *TSH* concentrations and their respective setpoints regarding the *L*-*T*_4_ monotherapy and increased *T*_3_ fluctuations regarding the *L*-*T*_3_/*L*-*T*_4_ combined therapy. On the other hand, when the patient has a CC genotype leading to a higher activity of D1 (here considered by *G*_*D*1_^′​′^=1.1*G*_*D*1_), there is no difference in the outcome of both therapies. There is enough endogenously produced *T*_3_ such that the setpoint of healthy individuals is reached even for the *L*-*T*_4_ monotherapy. The intrathyroidal production of *T*_3_ (that cannot be replaced by an *L*-*T*_4_ monotherapy) is compensated by the increased D1 activity. In contrast to the *L*-*T*_4_ monotherapy, the *L*-*T*_3_/*L*-*T*_4_ combined therapy goes along with high fluctuations of *T*_3_ during the first days of therapy. Furthermore, the simulation results for *G*_*D* 1_^′​′​′^=1.2*G*_*D* 1_ (which is another simple exemplary modeling of two C-allele) show that both therapies reach the setpoint up to some small offset; the concentrations of *T*_3_ and TSH are slightly higher than their setpoints whereas the concentration of *T*_4_ remains slightly lower than the respective setpoint. Here, the intrathyroidal production of *T*_3_ is overcompensated by the increased activity of *D*1, explaining the opposite results of **Figure 2** (where a too low concentration of *T*_3_ and a too high concentration of *T*_4_ is observed). Therefore, an additional intake of *L*-*T*_3_ would lead to even higher concentrations of *T*_3_ which are not desired. Consequently, even though two medication inputs are available, it is impossible to reach the euthyroid setpoint. In **Figure 6D**, one can see that after day three no *L*-*T*_3_ is needed to remain at the steady state. Therefore, the *L*-*T*_3_/*L*-*T*_4_ combined therapy reduces to an *L*-*T*_4_ monotherapy. If one additionally prescribes *L*-*T*_3_ after day three, the *T*_3_ concentrations would be even higher which potentially leads to symptoms of hyperthyroidism.

In conclusion, our results suggest that in case of genotype AA regarding polymorphism rs2235544 of *DIO1*, an *L*-*T*_3_/*L*-*T*_4_ combined therapy can be beneficial, whereas an *L*-*T*_4_ monotherapy seems to be better suited (with respect to the achieved thyroid hormone concentration) in case of genotype CC concerning polymorphism rs2235544 of *DIO1*. However, clinical studies do not document a better response (in terms of symptoms) to an *L*-*T*_3_/*L*-*T*_4_ combined therapy when patients are affected by this polymorphism (26). This difference to our result could be explained by the hypotheses mentioned in (16) stating that the success of the *L*-*T*_3_/*L*-*T*_4_ combined therapy strongly depends on whether the *T*_3_ concentrations are brought to the upper reference range and on the residual thyroid function. Furthermore, the simulations of polymorphism rs225014 reveal that the *L*-*T*_3_/*L*-*T*_4_ combined therapy is better to treat hypothyroidism in terms of the treated steady-state hormone concentrations, if the *T*_3_ setpoint is set to the upper limit of the reference range of healthy individuals, compare **Figures 7**, **8** in the **Supplementary Material**. This result is in line with the study (26), where the authors document a better response to the *L*-*T*_3_/*L*-*T*_4_ combined therapy for patients affected by the considered genotype. Once again, these results underline the relevance of the hypothesis that the *L*-*T*_3_/*L*-*T*_4_ combined therapy is successful if the *T*_3_ concentrations are brought to the upper reference range of healthy individuals because the *L*-*T*_3_/*L*-*T*_4_ combined therapy does not yield to an improved outcome in terms of the treated steady-state hormone concentrations, if the *T*_3_ setpoint remains unchanged. In any case, the benefit of the *L*-*T*_3_/*L*-*T*_4_ combined therapy might depend strongly on genetic variants. Such genetic variants could play a crucial role in explaining why some studies document an advantage of the *L*-*T*_3_/*L*-*T*_4_ combined therapy over the *L*-*T*_4_ monotherapy and others not as also suggested in (13).

### 4.3 Frequency of Intake

Before going into detail regarding the optimal *L*-*T*_3_ intake frequency in order to reach stable *T*_3_ concentrations, we briefly want to comment on the impact of the *L*-*T*_4_ intake frequency on the course of the *T*_4_ concentrations. These are known to be stable in the case of an *L*-*T*_4_ monotherapy for one daily intake (13). The simulations of the *L*-*T*_4_ monotherapy emphasize this observation, compare **Figures 2**, **7** as well as **Table 1**. Two or three daily medication intakes lead to reduced fluctuations of *T*_4_ as shown in **Table 1**. However, since many patients feel well with one daily intake of *L*-*T*_4_, there is potentially no need in increasing the intake frequency although the fluctuations would decrease. Furthermore, the fluctuations of the *T*_4_ concentrations are similar concerning the *L*-*T*_3_/*L*-*T*_4_ combined therapy. This is due to the similar dosages of *L*-*T*_4_ regarding the *L*-*T*_4_ monotherapy and the *L*-*T*_3_/*L*-*T*_4_ combined therapy. In addition, the fluctuations of the *T*_4_ concentrations are similar in the transient phase and in the steady state, compare *ΔT*_4_ in **Table 1** for one daily intake. The long half-life of approximately one week leads to stable hormone concentrations, such that the fluctuations are not impacted considerably by the different medication dosages occurring in the transient phase and in the steady state.

Considering the *T*_3_ concentrations in **Figures 2** and **7** as well as in **Table 1**, one can see that the *L*-*T*_4_ monotherapy leads to stable *T*_3_ concentrations, even in the case of one daily intake. Regarding the *L*-*T*_3_/*L*-*T*_4_ combined therapy, the frequency of the intake of *L*-*T*_3_ influences substantially the fluctuations of the *T*_3_ concentrations, compare **Figures 3**, **8**, as well as **Table 1**. As expected, a higher intake frequency of *L*-*T*_3_ results in lower fluctuations of the *T*_3_ concentrations. However, a higher intake frequency is also less convenient for patients, especially since these therapies are often life-long therapies. Given the numbers reported in **Table 1**, a good trade-off could be to consider two daily intakes in case of the *L*-*T*_3_/*L*-*T*_4_ combined therapy.

## 5 Conclusion

In this work, we design an MPC for the pituitary-thyroid feedback loop in order to develop optimal thyroid hormone replacement strategies. By means of this approach, an improvement of the current trial-and-error process of prescribing thyroid replacement hormones is possible. Furthermore, the simulations indicate that the *L*-*T*_3_/*L*-*T*_4_ combined therapy is, in general, slightly superior to treat hypothyroidism compared to the *L*-*T*_4_ monotherapy. In case of genotype CC concerning polymorphism rs2235544 of gene DIO1, the *L*-*T*_4_ monotherapy shows better results than the *L*-*T*_3_/*L*-*T*_4_ combined therapy. In turn, regarding genotype AA of polymorphism rs2235544, the *L*-*T*_3_/*L*-*T*_4_ combined therapy seems to be more suitable. Furthermore, we observe an improved outcome (in terms of treated hormone concentrations) of the *L*-*T*_3_/*L*-*T*_4_ combined therapy when patients are affected by genotype CC of polymorphism rs225014 in gene *DIO2* for a *T*_3_ setpoint at the upper limit of reference range of healthy individuals. The dependence of the results on genetic variants might explain the conflicting results of existing clinical studies focusing on a comparison of both therapies. In order to reach stable *T*_3_ concentrations and a sufficiently convenient therapy, a good trade-off for an *L*-*T*_3_/*L*-*T*_4_ combined therapy is to consider two daily drug intakes.

## Data Availability Statement

The original contributions presented in the study are included in the article/**Supplementary Material**. Further inquiries can be directed to the corresponding author.

## Author Contributions

TW drafted the manuscript and performed the calculations/simulations with Matlab/Simulink, under the supervision of MM and with input from JD. All authors contributed to the article and approved the submitted version.

## Funding

This project has received funding from the European Research Council (ERC) under the European Union’s Horizon 2020 research and innovation programme (grant agreement No 948679).

## Conflict of Interest

JD received funding and personal fees by Sanofi-Henning, Hexal AG, Bristol-Myers Squibb, and Pfizer, and is co-owner of the intellectual property rights for the patent “System and Method for Deriving Parameters for Homeostatic Feedback Control of an Individual” (Singapore Institute for Clinical Sciences, Biomedical Sciences Institutes, Application Number 201208940-5, WIPO number WO/2014/088516).

The remaining authors declare that the research was conducted in the absence of any commercial or financial relationships that could be construed as a potential conflict of interest.

## Publisher’s Note

All claims expressed in this article are solely those of the authors and do not necessarily represent those of their affiliated organizations, or those of the publisher, the editors and the reviewers. Any product that may be evaluated in this article, or claim that may be made by its manufacturer, is not guaranteed or endorsed by the publisher.

## Footnotes

1 The parameters G_D1_ and G_T3_ are determined using thyroid hormone measurements from healthy individuals (considering men and women) with a mean age of approximately 25 years, a mean body weight of approximately 70kg, and a BMI of approximately 22kg/m^2^, compare also (19, 20).

2 The considered generic hypothyroid patients have the same characteristics with respect to sex, age, body weight, and BMI as the considered healthy individuals, compare footnote 1.
