# Supplementary material for: Optimal Hormone Replacement Therapy in Hypothyroidism - A Model Predictive Control Approach
Source: Front Endocrinol (Lausanne). 2022 Jun 24;13:884018. doi: 10.3389/fendo.2022.884018 (PMC9263720; doi:10.3389/fendo.2022.884018)
Supplement: Supplementary file 1 [file DataSheet_1.pdf]

Supplementary Material:
Optimal hormone replacement therapy in hypothyroidism - a model predictive control approach

Tobias M. Wolff *, Johannes W. Dietrich and Matthias A. Müller

Correspondence*:
Tobias M. Wolff
wolff@irt.uni-hannover.de

S1 SYSTEM'S DIFFERENTIAL EQUATIONS

Here, we introduce the six nonlinear differential equations on which the mathematical model is based. They describe the cause-effect relations of the pituitary-thyroid feedback loop. In fact, Figure 1 of the main text is an illustration of equations (S1)-(S11). The numerical parameter values are given in Section S4. They have been derived experimentally, are known quantities, or have been fitted to real thyroid hormone measurements. The differential equations are

$$\begin{aligned}\frac{dT_{4,th}}{dt}(t) =&\alpha_{th}\Bigg(G_T\frac{TSH(t)}{TSH(t)+D_T} - G_{MCT8}\frac{T_{4,th}(t)}{K_{MCT8}+T_{4,th}(t)} - G_{D1}\frac{T_{4,th}(t)\frac{TSH(t)}{TSH(t)+k_{Dio}}}{T_{4,th}(t)\frac{TSH(t)}{TSH(t)+k_{Dio}}+K_{M1}}\\ &- G_{D2}\frac{T_{4,th}(t)\frac{TSH(t)}{TSH(t)+k_{Dio}}}{T_{4,th}(t)\frac{TSH(t)}{TSH(t)+k_{Dio}}+K_{M2}}\Bigg) - \beta_{th}T_{4,th}(t) \end{aligned} \tag{S1}$$

$$\frac{dT_4}{dt}(t) =\alpha_T\Big(G_{MCT8}\frac{T_{4,th}(t)}{K_{MCT8}+T_{4,th}(t)} + u_{L\text{-}T_4}(t)\Big) - \beta_T T_4(t) \tag{S2}$$

$$\begin{aligned}\frac{dT_{3p}}{dt}(t) =&\alpha_{31}\Bigg(G_{D1}\frac{FT_4(t)}{FT_4(t)+K_{M1}} + G_{D2}\frac{FT_4(t)}{FT_4(t)+K_{M2}} + G_{D1}\frac{T_{4,th}(t)\frac{TSH(t)}{TSH(t)+k_{Dio}}}{T_{4,th}(t)\frac{TSH(t)}{TSH(t)+k_{Dio}}+K_{M1}}\\ &+ G_{D2}\frac{T_{4,th}(t)\frac{TSH(t)}{TSH(t)+k_{Dio}}}{T_{4,th}(t)\frac{TSH(t)}{TSH(t)+k_{Dio}}+K_{M2}} + G_{T3}\frac{TSH(t)}{D_T+TSH(t)} + u_{L\text{-}T_3}(t)\Bigg) - \beta_{31}T_{3p}(t) \end{aligned} \tag{S3}$$

$$\frac{dT_{3c}}{dt}(t) = \alpha_{32} G_{D2} \frac{FT_4(t)}{FT_4(t) + K_{M2}} - \beta_{32} T_{3c}(t) \tag{S4}$$

$$\frac{dTSH}{dt}(t) = \frac{\alpha_S G_H TRH}{(TRH + D_H)(1 + S_S \frac{TSH_z(t)}{TSH_z(t)+D_S})(1 + L_S G_R \frac{T_{3N}(t)}{T_{3N}(t)+D_R})} - \beta_S TSH(t) \tag{S5}$$

$$\frac{dTSH_z}{dt}(t) = \frac{\alpha_{S2} G_H TRH}{(TRH + D_H)(1 + S_S \frac{TSH_z(t)}{TSH_z(t)+D_S})(1 + L_S G_R \frac{T_{3N}(t)}{T_{3N}(t)+D_R})} - \beta_{S2} TSH_z(t) \tag{S6}$$

with the relationships

$$FT_3 = T_{3p} \frac{1}{1 + K_{30} TBG} \tag{S7}$$

$$FT_4 = T_4 \frac{1}{1 + K_{41} TBG + K_{42} TBPA} \tag{S8}$$

$$T_{3N} = T_{3c} \frac{1}{1 + K_{31} IBS} \tag{S9}$$

$$u_{L\text{-}T_3}(t) = k_{33} \frac{k_{13}}{k_{13} - (k_{23} + k_{33})} \sum_{l=0}^{N-1} m_{D3,l} \left(e^{-(k_{23}+k_{33})(t - lt_l)} - e^{-k_{13}(t - lt_l)} \right) \tag{S10}$$

$$u_{L\text{-}T_4}(t) = k_{34} \frac{k_{14}}{k_{14} - (k_{24} + k_{34})} \sum_{l=0}^{N-1} m_{D4,l} \left(e^{-(k_{24}+k_{34})(t - lt_l)} - e^{-k_{14}(t - lt_l)} \right). \tag{S11}$$

In the following, we describe in more detail the equations for $u_{L\text{-}T_3}$ (S10) and $u_{L\text{-}T_4}$ (S11), since these are introduced in this work. For more information about the remaining differential equations, the reader is referred to (1, Supplementary Material) and to (2, 3). Equations (S10) and (S11) are based on the linear dissolution and absorption model of thyroid replacement hormones from (4, eqs. (B.1), (B.2)). The concept of the model is the following: first, a thyroid replacement hormone enters the gut in an undissolved form. Second, this undissolved medication dissolves with rate k_1 and, thus, is available in a dissolved form. Third, rate k_2 considers the excretion of the medication and finally k_3 denotes the absorption rate from the gut into the blood (and therefore into the pituitary-thyroid feedback loop). The solution of the oral medication intake model is

$$u_{L\text{-}T_i}(t) = k_{3i} \frac{k_{1i}}{k_{1i} - (k_{2i} + k_{3i})} m_{Di} \left(e^{-(k_{2i}+k_{3i})t} - e^{-k_{1i}t} \right) \tag{S12}$$

for $i = \{3, 4\}$ (compare (4, eq. (B.7))). This analytical function describes the time-dependent absorption of the medication. Over the period of several days, more than one medication is taken in, which makes it necessary to consider the effect of the sum of the medication intakes. Since the dissolution and absorption model is linear, we can sum up the absorption rates of the different medication intakes to get $u_{L\text{-}T_3}(t)$ (S10) and $u_{L\text{-}T_4}(t)$ (S11). The variables m_{D3} and m_{D4} stand for the medication dosages L-T_3 and L-T_4, respectively. The sum index N denotes the total number of medication intakes, which differs whether we consider one, two, or three daily medication intakes. In the case of one daily intake, N is equal to the number of considered days, e.g., in Figure 2 of the main article, $N = 15$. The variable t_l denotes the time interval between two medication intakes: for one daily intake $t_l = 24\text{h}$, for two daily intakes $t_l = 12\text{h}$, and for three daily intakes $t_l = 8\text{h}$.

S2 DETAILED DESCRIPTION OF THE MPC

As mentioned in the main part, we implement an MPC to develop optimal thyroid hormone replacement strategies. The procedure of an MPC is the following: at each time t, the state x is measured and an optimal control problem is solved. Next, the first part of the optimal input is applied to the system. At the next sampling instant, this process is repeated. The general MPC setting for nonlinear systems in continuous time is as follows (5): at time t, solve the following optimization problem

$$\min_{\overline{u}(\cdot;t)} J\big(x(t), \overline{u}(\cdot;t)\big) \tag{S13}$$

with

$$J(x(t), \overline{u}(\cdot;t)) = \int_t^{t+T} L\big(\overline{x}(\tau;t), \overline{u}(\tau;t)\big) d\tau \tag{S14}$$

$$= \int_t^{t+T} ||\overline{x}(\tau;t) - x_s||_Q^2 + ||\overline{u}(\tau;t) - u_s||_R^2 \, d\tau. \tag{S15}$$

subject to constraints

$$\dot{\overline{x}} = f(\overline{x}, \overline{u}) \qquad \overline{x}(t;t) = x(t) \tag{S16}$$

$$\overline{u}(\tau;t) \in \mathcal{U} \tag{S17}$$

$$\overline{x}(\tau;t) \in \mathcal{X} \tag{S18}$$

for $\tau \in [t, t+T]$. Here, J denotes the objective function that has to be minimized, f the system dynamics (in our case the right hand side of (S1)-(S6)), and T the horizon length (here five days). The states of the system are denoted by x. In our application, the states are $x = \begin{bmatrix} T_{4,th} & T_4 & T_{3p} & T_{3c} & TSH & TSH_z \end{bmatrix}^\top$. The inputs are $u = \begin{bmatrix} m_{D3} & m_{D4} \end{bmatrix}^\top$, the medication dosages of L-T_3 and L-T_4, respectively. The stage cost is denoted by L and the weighting matrices for the states and the inputs by Q and R, respectively. Constraint (S16) is necessary such that the dynamics of the system are respected and the first predicted element of the state corresponds to the given (measured) starting point. Next, the expressions (S17) and (S18) denote the input and state constraints. Using an MPC, it is possible to specify input and state constraints that are respected by the controller.

In this work, we chose the following numerical values

$$Q = \begin{bmatrix} 1 & 0 & 0 & 0 & 0 & 0 \\ 0 & 1000 & 0 & 0 & 0 & 0 \\ 0 & 0 & 1000 & 0 & 0 & 0 \\ 0 & 0 & 0 & 1 & 0 & 0 \\ 0 & 0 & 0 & 0 & 1000 & 0 \\ 0 & 0 & 0 & 0 & 0 & 1 \end{bmatrix}, \quad R = 0, \tag{S19}$$

$$\mathcal{X} := \{x \in \mathbb{R}^6 | x \geq 0\}, \quad \mathcal{U} := \{u \in \mathbb{R} | 0 \leq u \leq u_{\max}\}. \tag{S20}$$

The weighting matrix Q penalizes deviations of the states T_4, T_3 and TSH from their respective equilibrium points x_s. The deviations of the remaining states from their equilibrium points do not influence the cost function considerably. The choice of R guarantees that the input, i.e., the height of the medication dosages does not influence the cost function. In the here considered application, our objective is not, e.g., to use as little medication as possible, but to bring the hormone concentrations to their desired setpoints. In addition, we use $u_s = 0$. The state and input constraints ($\mathcal{X}$, $\mathcal{U}$) summarize our physical information about the system. Neither the hormone concentrations nor the medication dosages can have negative values. The medication dosages, i.e., the inputs to the system are limited. Regarding the dosages of L-T_4, the maximal daily dosage is set to 400 µg and regarding L-T_3 to 30 µg, as suggested in (6). In the case of two (three) daily intakes, every intake is limited by 200 µg (133 µg) regarding L-T_4 and 15 µg (10 µg) regarding L-T_3. If we considered a higher daily limit regarding the L-T_4 and L-T_3 dosages, the hormone concentrations would reach their steady states faster and vice versa. The implementation of the MPC is realized by means of a discretization using the single shooting method (7).

S3 ADDITIONAL RESULTS

S3.1 Impact of the Secretory Capacity of the Thyroid G_T

As mentioned in the main part, different numerical values of G_T lead to the same qualitative results. Here, we reinforce this claim by simulation results in which the numerical value of G_T is chosen to be 5 % and 20 % of the numerical value of an euthyroid individual. In Figure 1, the results for $G_T' = 0.05G_T$ are illustrated and in Figure 2, the results for $G_T'' = 0.20G_T$ are shown.

In these figures, it becomes clear that a different value of G_T still leads to the same qualitative results. Figures 1 and 2 show that an adapted numerical value of G_T mainly impacts the medication dosages of L-T_3 and L-T_4. Obviously, a higher value of G_T (implying an increased secretory capacity of the thyroid) leads to higher endogenous production of T_3 and T_4. Therefore, less medication is needed in order to normalize the T_4 and the T_3 concentrations explaining the relatively low dosages of L-T_4 in Figure 2 and the relatively high dosages of L-T_4 in Figure 1.

In addition, the simulation results reveal that the impact of the TSH-T_3 shunt (describing the intrathyroidal T_3 production, compare (3)) is visible for all values of G_T that are used here. In general, the impact of the TSH-T_3 shunt is higher for smaller values of G_T, meaning that the offset between the setpoint and the hormone concentrations is higher for smaller values of G_T in case of the L-T_4 monotherapy, see Figures 1 **A**, **B** and 2 **A**, **B**. This is qualitatively the same observation as seen in Figure 2 of the main article. Once again, this offset cannot be seen in the case of the L-T_3/L-T_4 combined therapy (see Figures 1 **C**, **D** and 2 **C**, **D**).

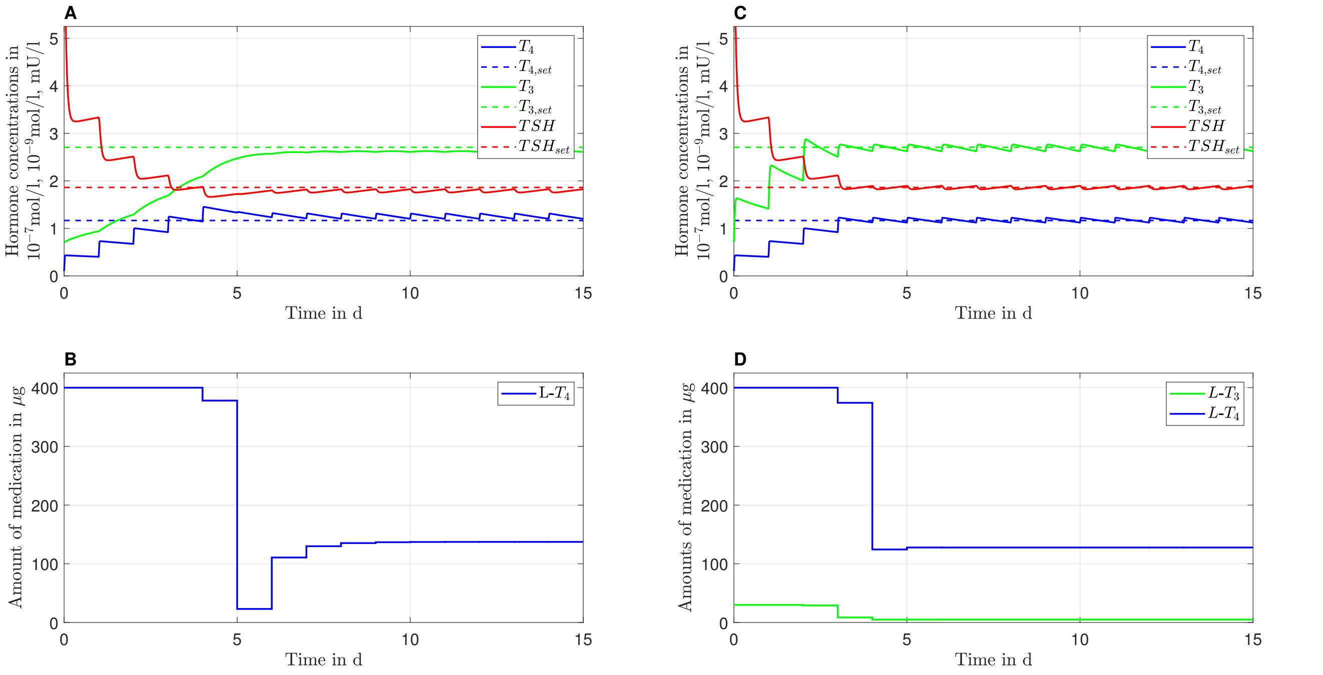

Figure 1. Simulation results of an L-T_4 monotherapy (**A**, **B**) and an L-T_3/L-T_4 combined therapy (**C**, **D**) for $G'_T = 0.05G_T$.

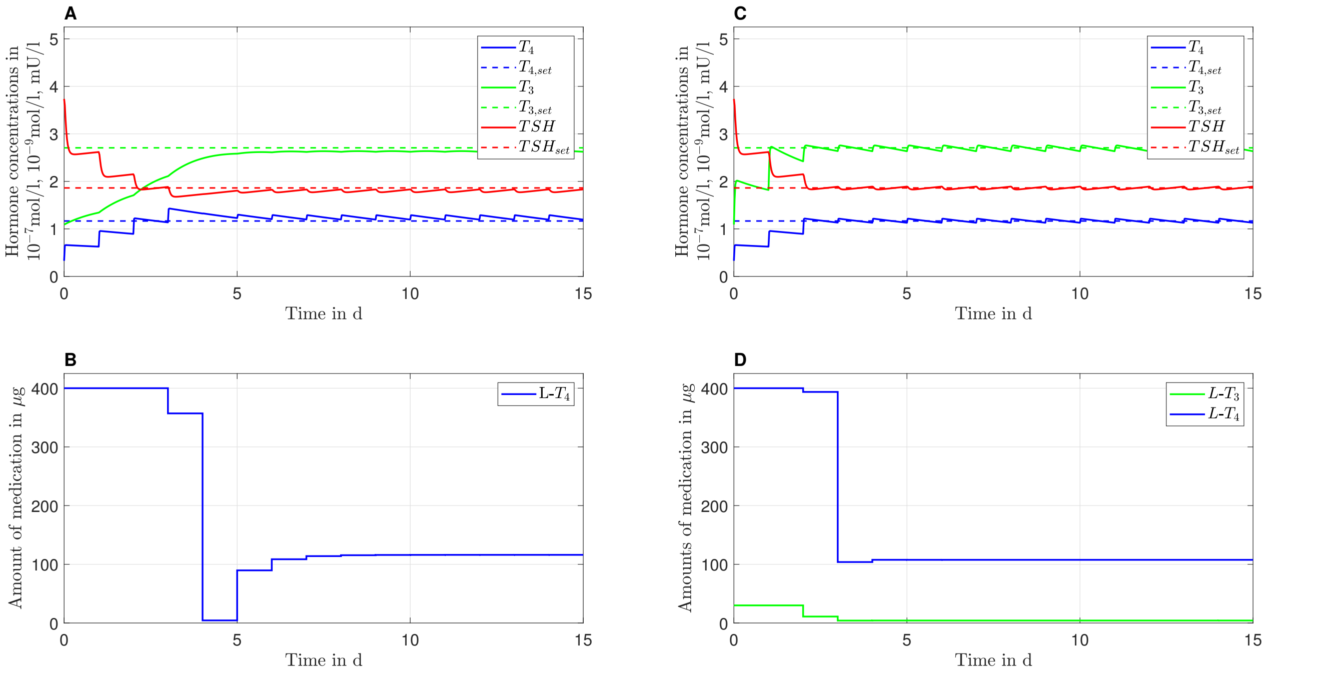

Figure 2. Simulation results of an L-T_4 monotherapy (**A**, **B**) and an L-T_3/L-T_4 combined therapy (**C**, **D**) for $G''_T = 0.20G_T$.

S3.2 Impact of Different Cost Functions

In this subsection, the results of an L-T_4 monotherapy and an L-T_3/L-T_4 combined therapy using different cost functions are illustrated. The numerical value of R remains unchanged ($R = 0$) throughout this section. In Figure 3, the impact of a cost function mainly penalizing the deviations of T_3 to its euthyroid setpoint is illustrated for an L-T_4 monotherapy and an L-T_3/L-T_4 combined therapy. The corresponding weighting matrix Q' is defined as

$$Q' = \begin{bmatrix} 1 & 0 & 0 & 0 & 0 & 0 \\ 0 & 1 & 0 & 0 & 0 & 0 \\ 0 & 0 & 1000 & 0 & 0 & 0 \\ 0 & 0 & 0 & 1 & 0 & 0 \\ 0 & 0 & 0 & 0 & 1 & 0 \\ 0 & 0 & 0 & 0 & 0 & 1 \end{bmatrix}. \quad \text{(S21)}$$

Furthermore, we perform simulations considering a cost function mainly penalizing the deviations of T_4 to its euthyroid setpoint. The results are illustrated in Figure 4. The corresponding weighting matrix Q'' is defined as

$$Q'' = \begin{bmatrix} 1 & 0 & 0 & 0 & 0 & 0 \\ 0 & 1000 & 0 & 0 & 0 & 0 \\ 0 & 0 & 1 & 0 & 0 & 0 \\ 0 & 0 & 0 & 1 & 0 & 0 \\ 0 & 0 & 0 & 0 & 1 & 0 \\ 0 & 0 & 0 & 0 & 0 & 1 \end{bmatrix}. \quad \text{(S22)}$$

In Figure 5, the simulation results of a cost function mainly penalizing the deviations from TSH to its euthyroid setpoint are illustrated. The corresponding matrix Q''' is defined as

$$Q''' = \begin{bmatrix} 1 & 0 & 0 & 0 & 0 & 0 \\ 0 & 1 & 0 & 0 & 0 & 0 \\ 0 & 0 & 1 & 0 & 0 & 0 \\ 0 & 0 & 0 & 1 & 0 & 0 \\ 0 & 0 & 0 & 0 & 1000 & 0 \\ 0 & 0 & 0 & 0 & 0 & 1 \end{bmatrix}. \quad \text{(S23)}$$

The following comments regarding the simulation results are in order. First, in Figure 3 **A**, **B**, one can see that a normalization of the T_3 concentrations goes along with considerably high concentrations of T_4. This observation is also visible in Figure 2 of the main article. However, due to the adapted cost function, which mainly penalizes the deviations of T_3 to its euthyroid setpoint in Figure 3 **A**, **B**, the MPC focuses mainly on the normalization of the T_3 concentrations and not on a joint normalization of the T_3, T_4, and TSH concentrations as it is the case in Figure 2 of the main article. Therefore, the offset of T_4 to its setpoint is higher when the adapted cost function using Q' is used. In (8), the authors also report that an L-T_4 monotherapy usually leads to high concentrations of T_4.

Interestingly, the L-T_3/L-T_4 combined therapy goes along with very small dosages of L-T_3. L-T_3 has a short half-life of approximately one day. Therefore, an intake of L-T_3 implies high fluctuations of T_3 (as visible in Figure 3 of the main article. Here, these fluctuations lead to higher costs compared to high

dosages of L-T_4 that can also normalize the T_3 concentrations (via D1 and D2). The difference compared to the results documented in Figure 3 of the main text can be explained by means of a different choice of the weighting matrix Q'.

In conclusion, both types of therapy reach the desired euthyroid concentration of T_3. Therefore, if one is only interested in normal T_3 concentrations, one should choose the L-T_4 monotherapy, due to its simplicity and the low fluctuations of the T_3 concentrations.

In Figure 4, the simulation results of an L-T_4 monotherapy and an L-T_3/L-T_4 combined therapy are illustrated for Q'' (penalizing the deviations of T_4 to its euthyroid setpoint). Regarding the L-T_4 monotherapy (compare Figure 4 **A**, **B**), the T_4 (and the TSH) concentration normalizes, whereas the concentration of T_3 remains much lower than its euthyroid setpoint. An increased concentration of T_3 could only be realized by increasing the dosage of L-T_4. However, this would inherently lead to a higher concentration of T_4 and thus resulting in higher costs.

In case of the L-T_3/L-T_4 combined therapy (see Figure 4 **C**, **D**), not only the T_4 and the TSH concentrations normalize, but also the T_3 concentrations. This can be explained by the additional intake of T_3. In contrast to the L-T_4 monotherapy, one can simply take in L-T_3 to normalize the T_3 concentrations without inherently increasing the T_4 concentrations (and thus increasing the costs).

Finally, we comment on the simulation results using Q''', which mainly penalizes deviations of the TSH concentrations to its euthyroid setpoint. In that case (compare Figure 5 **A**, **B**), the results are similar to the ones, in which deviations of T_4 to its euthyroid setpoint are penalized. This is due to the fact that FT_4 regulates the TSH production (via the pituitary, compare the upper part of Figure 1 of the main article). Since no parameter in the pituitary is changed in our model of hypothyroidism (but only the secretory capacity G_T of the thyroid), the relationship between T_4 and TSH remains unchanged. Consequently, a normalization of T_4 necessarily goes along with a normalization of TSH and vice versa when considering hypothyroidism in the mathematical model.

S3.3 Impact of Different Bioavailabilities regarding T_3 and T_4

In this subsection, we consider different bioavailabilities concerning the parameter fit (k_{1i}, k_{2i}, k_{3i}) of the oral medication intake model. As mentioned in the main part, the bioavailabilities of L-T_3 and L-T_4 depend on the thyroid state (9). In contrast to all of the previously shown simulations, we here apply the bioavailabilities of hypothyroid patients and use once again a least-squares fit to identify the parameters k_{1i}, k_{2i}, and k_{3i}. In Figure 6, the results of an L-T_4 monotherapy (**A**, **B**) and an L-T_3/L-T_4 combined therapy (**C**, **D**) are illustrated for one daily intake, without genetic variant and for a secretory capacity of the thyroid which corresponds to 10 % of the one of healthy individuals.

The general course of the hormone concentrations in both therapies does not change compared to the results exploiting the bioavailabilities of euthyroid individuals (see Figures 3, 4 of the main article, and Figure 6). Note that the L-T_4 dosages (shown in Figure 6) regarding the L-T_4 monotherapy and the L-T_3/L-T_4 combined therapy are lower compared to the dosages seen for the bioavailabilities of euthyroid controls, compare Figures 2 and 3 of the main article. This is due to the higher bioavailability of T_4 in the case of hypothyroid patients (84 %) compared to the bioavailability of euthyroid controls (65.5 %). The implication of a higher bioavailability is that less L-T_4 needs to be taken in to reach the euthyroid setpoint of healthy individuals. Since the bioavailability of L-T_3 remains approximately the same (euthyroid controls: 78.2 %, hypothyroid patients: 76.6 %), the L-T_3 dosages do not change substantially.

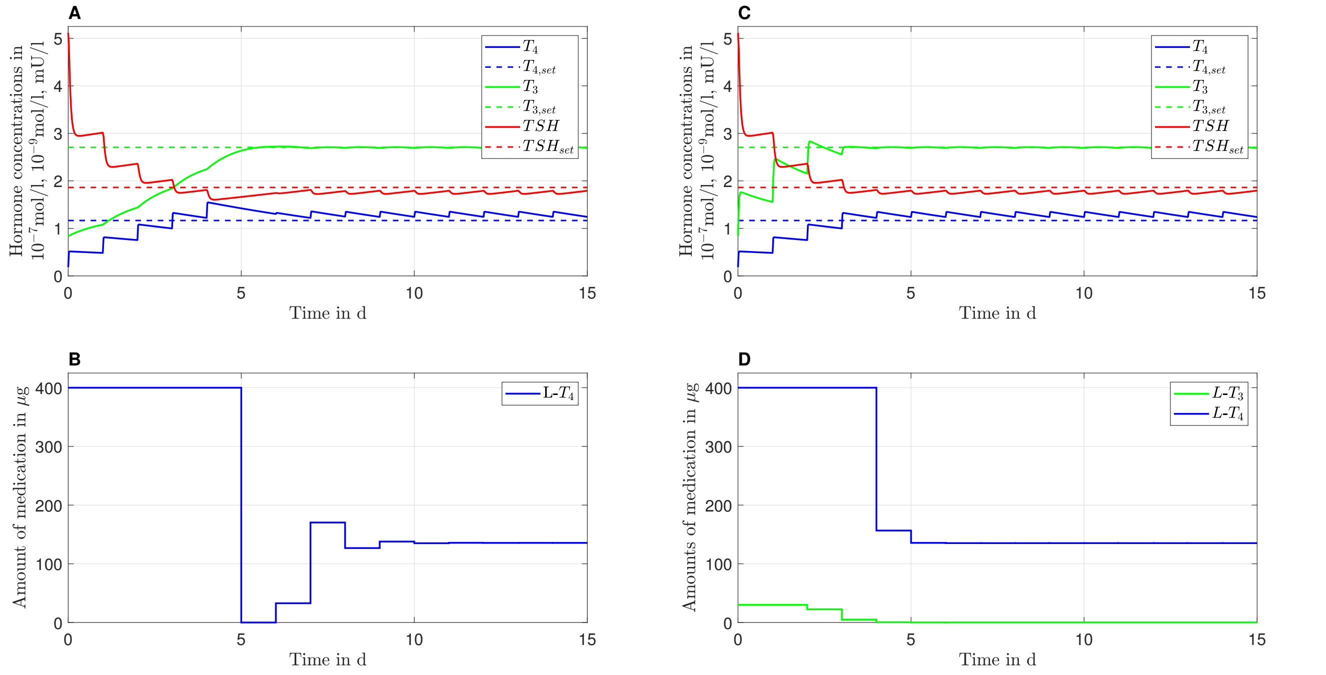

Figure 3. Simulation results of an L-T_4 monotherapy (**A**, **B**) and an L-T_3/L-T_4 combined therapy (**C**, **D**) for Q' (penalizing deviations of T_3 from its desired euthyroid setpoint).

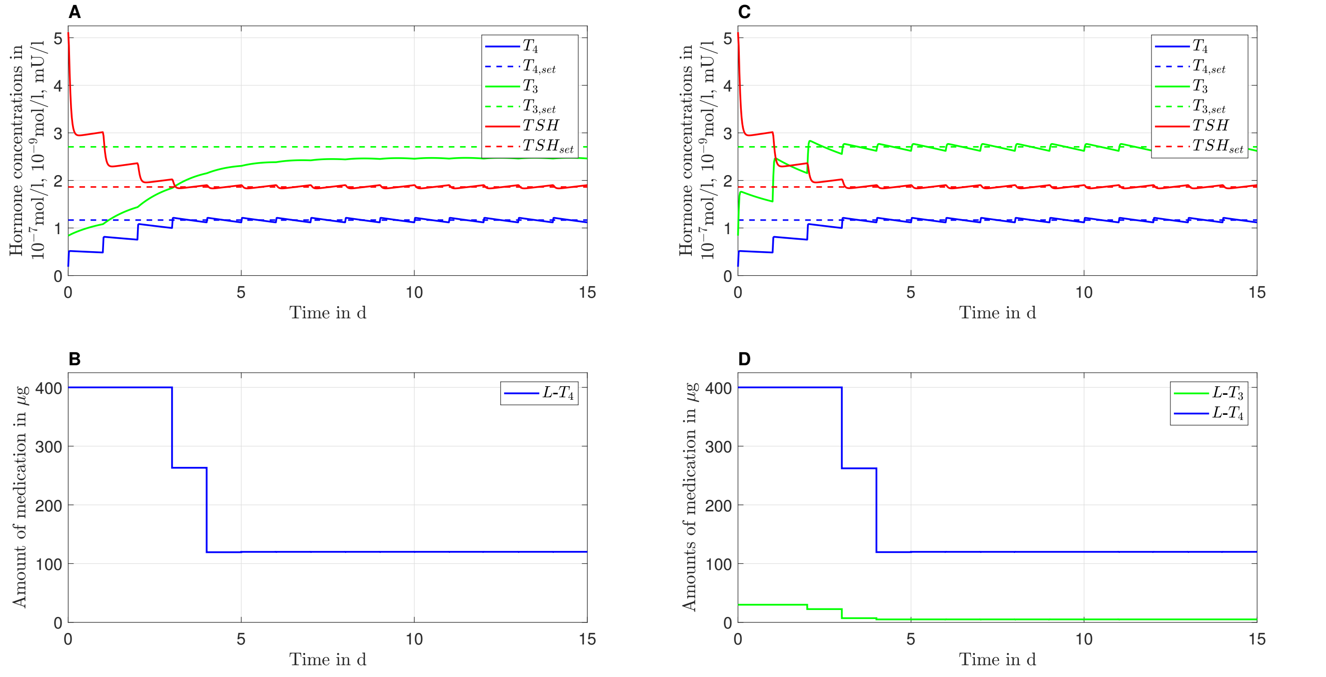

Figure 4. Simulation results of an L-T_4 monotherapy (**A**, **B**) and an L-T_3/L-T_4 combined therapy (**C**, **D**) for Q'' (penalizing deviations of T_4 from its desired euthyroid setpoint).

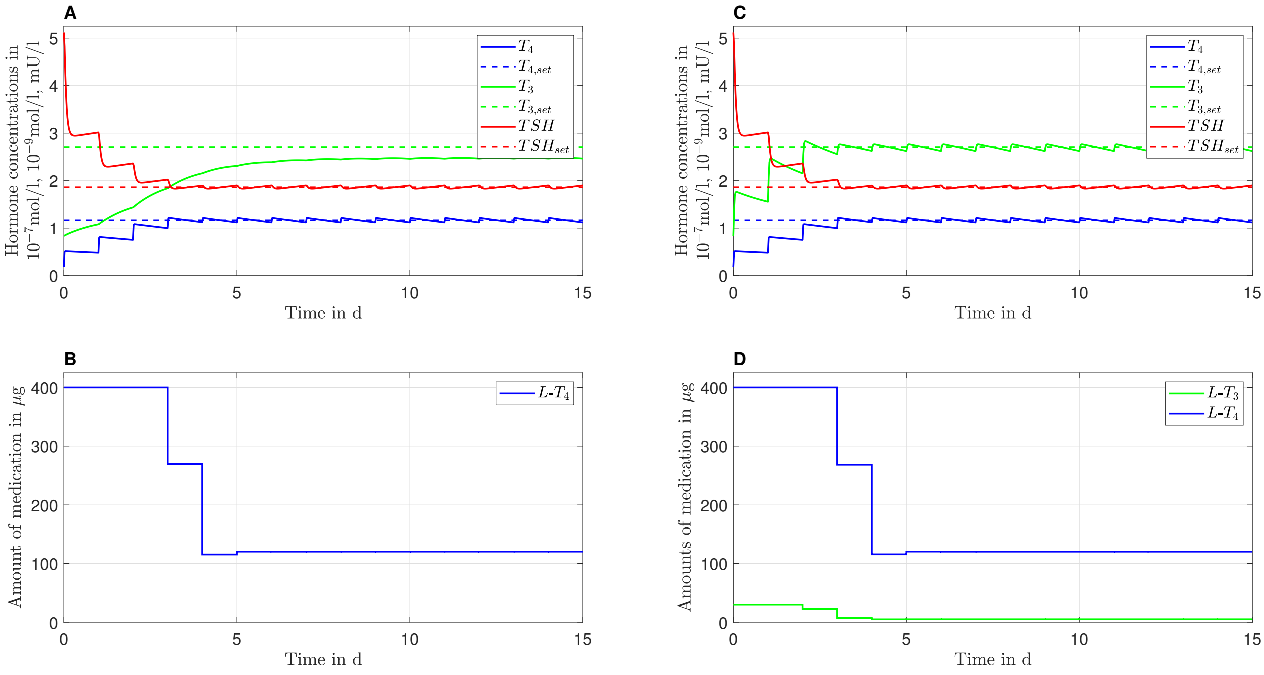

Figure 5. Simulation results of an L-T_4 monotherapy (**A**, **B**) and an L-T_3/L-T_4 combined therapy (**C**, **D**) for Q''' (penalizing deviations of TSH from its desired euthyroid setpoint).

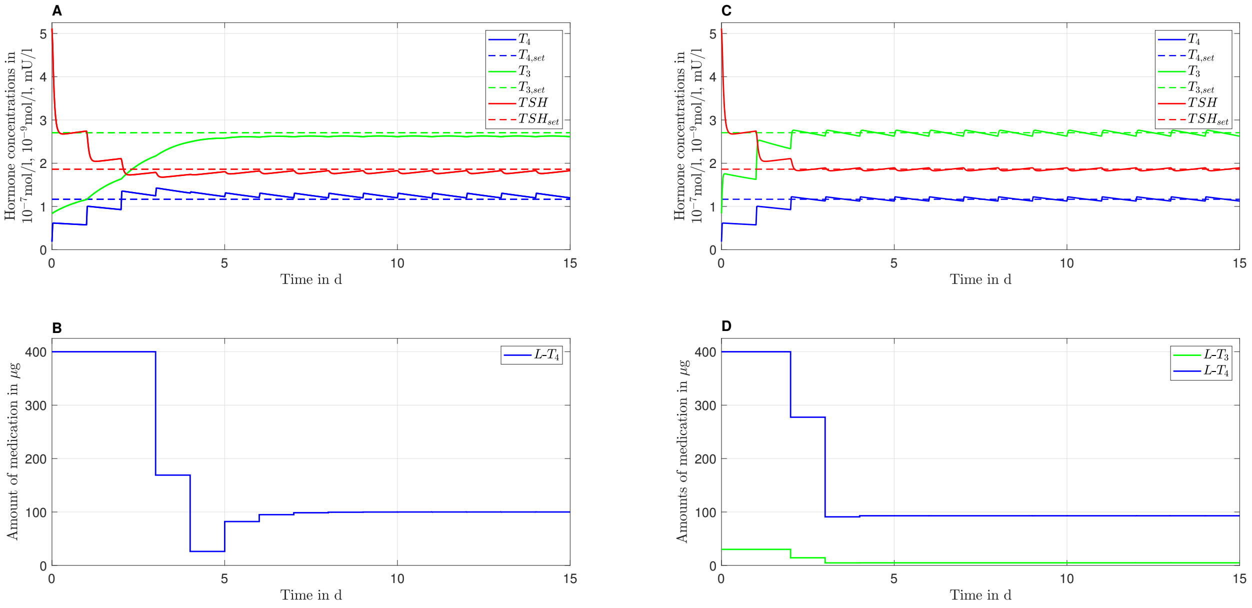

Figure 6. Simulation results of an L-T_4 monotherapy (**A**, **B**) and an L-T_3/L-T_4 combined therapy (**C**, **D**) for the bioavailabilities of hypothyroid patients as documented in (9).

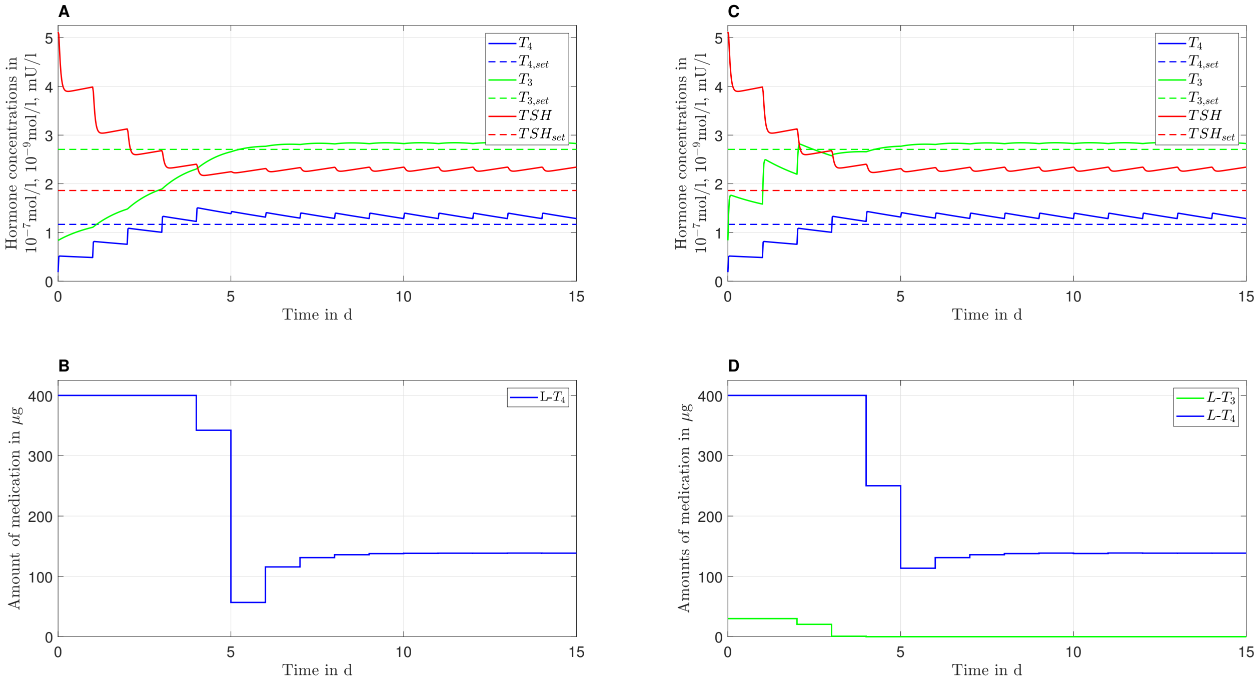

Figure 7. Simulation results of an L-T_4 monotherapy (**A**, **B**) and an L-T_3/L-T_4 combined therapy (**C**, **D**) for the CC genotype of polymorphism rs225014 considered by reducing G_{D2} to $G'_{D2} = 0.6G_{D2}$.

S3.4 Impact of the CC Genotype of Polymorphism rs225014

As mentioned in the main part, we here show the results for a modeling of the CC genotype of polymorphism rs225014 ($G'_{D2} = 0.6G_{D2}$). These results are illustrated in Figure 7. In this figure, one can see that there is virtually no difference in the steady-state hormone concentrations between the L-T_4 monotherapy and the L-T_3/L-T_4 combined therapy. Solely the setpoint regarding the T_3 concentrations is reached faster for the L-T_3/L-T_4 combined therapy. Since $D2$ converts T_4 into central T_3 in the pituitary and since this central T_3 inhibits the TSH synthesis (compare Figure 1 of the main manuscript, block “pituitary”, and equation (S4)), a reduction of the G_{D2} parameter leads to a smaller inhibition of the TSH concentrations. Therefore, even though the T_4 concentrations in Figure 7 are relatively high, they do not lead to small TSH concentrations. The high T_3 concentrations can be explained by the peripheral conversion of T_4 into T_3. The T_4 concentrations are relatively high and in the employed model (S1) – (S11) the conversion of T_4 into T_3 in the periphery is mainly carried out by D1 (and not influenced substantially by D2 since $G_{D1} >> G_{D2}$). Thus, the resulting T_3 concentrations are relatively high.

In (10), the authors suggest that an L-T_3/L-T_4 combined therapy is successful, if the treated steady-state T_3 concentrations are at the upper range of healthy individuals. The simulation results for a T_3 setpoint at the upper limit of the reference range of healthy individuals are illustrated in Figure 8. In this case, one can see that the L-T_3/L-T_4 combined therapy leads to better steady-state hormone concentrations compared to the L-T_4 monotherapy.

Please find a discussion of these results in the main part in Section 4.2.

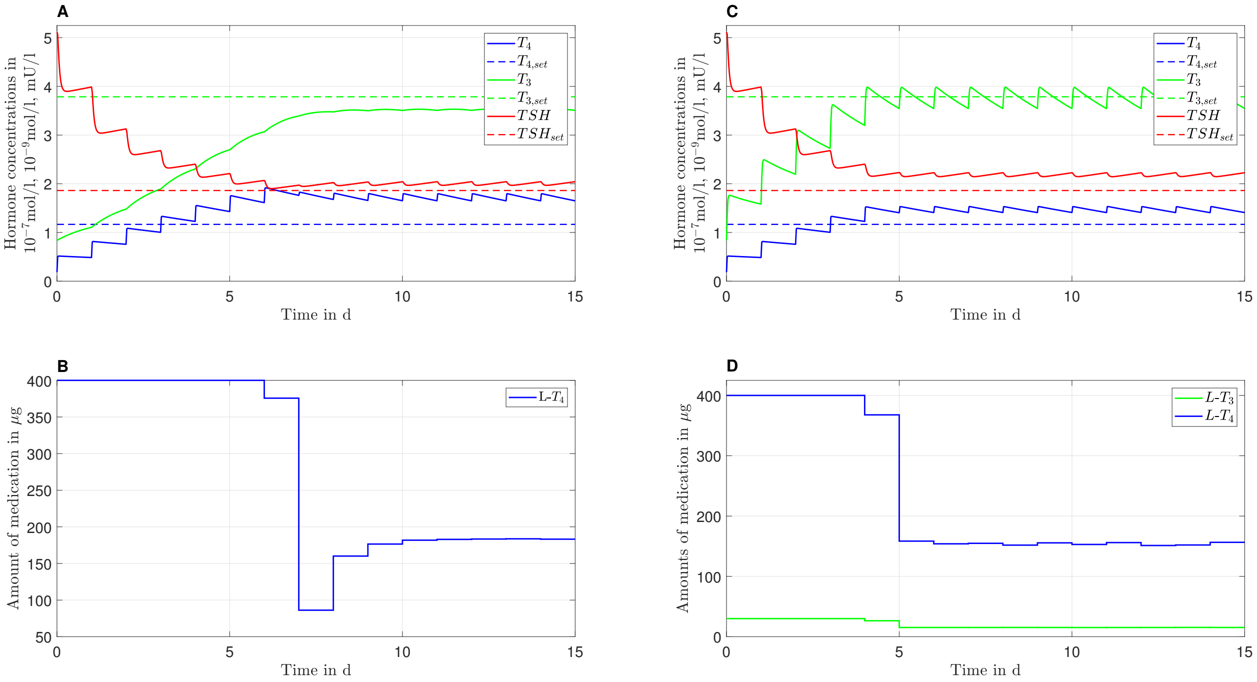

Figure 8. Simulation results of an L-T_4 monotherapy (**A**, **B**) and an L-T_3/L-T_4 combined therapy (**C**, **D**) for the CC genotype of polymorphism rs225014 considered by reducing G_{D2} to $G'_{D2} = 0.6G_{D2}$ and a T_3 setpoint at the upper limit of the reference range of healthy individuals.

S4 NUMERICAL PARAMETER VALUES

Symbol	Description	Value	Origin
TBG	Concentration of thyroxine-binding Globulin	$300\,\mathrm{nmol/l}$	(11)/ well known reference value
$TBPA$	Concentration of Transthyretin	$4.5\,\mu\mathrm{mol/l}$	(11)/ well known reference value
IBS	Concentration of intra-cellular T_3-binding substrate	$8\,\mu\mathrm{mol/l}$	Estimated from TBG-concentration, corrected for intra-cellular T_3-accumulation (according to values from (12))
TRH	TRH-concentration in hypophyseal portal system	$6.9\,\mathrm{nmol/s}$	(13)
G_H	Secretory capacity of the pituitary	$817\,\mathrm{mIU/s}$	Calculated according to (14) and (15)
D_H	Damping constant (EC_{50}) of TRH at the pituitary	$47\,\mathrm{nmol/s}$	(16)
α_S	Dilution factor for peripheral TSH	$0.4\,\mathrm{l}^{-1}$	Reciprocal value of the volume of distribution of $2.5\,\mathrm{l}$ (2)
β_S	Clearance exponent for peripheral TSH	$2.3 \cdot 10^{-4}\,\mathrm{s}^{-1}$	Calculated from plasma half-life of 50 min (17, 18)
L_S	Brake constant of long feedback	$1.68\,\mathrm{l/\mu mol}$	Calculated from clinical data of hyperthyroid patients (2)
G_T	Secretory capacity of thyroid gland	$3.4\,\mathrm{pmol/s}$	(18)
D_T	Damping constant (EC_{50}) at the thyroid gland	$2.75\,\mathrm{mIU/l}$	(19)
α_T	Dilution factor for T_4	$0.1\,\mathrm{l}^{-1}$	Reciprocal value of the volume of distribution (20)
β_T	Clearance exponent for T_4	$1.1 \cdot 10^{-6}\,\mathrm{s}^{-1}$	Calculated from plasma half-life of 7 days (20, 21)
K_{M1}	Dissociation constant of 5'-deiodinase type I	$500\,\mathrm{nmol/l}$	(20)
α_{31}	Dilution factor for peripheral T_3	$2.6 \cdot 10^{-2}\,\mathrm{l}^{-1}$	Reciprocal value of volume of distribution (20)
β_{31}	Clearance exponent for peripheral T_3	$8 \cdot 10^{-6}\,\mathrm{s}^{-1}$	Calculated from plasma half-life of 24 h (2)
G_{D2}	Maximum activity of 5'deiodinase type II	$4.3\,\mathrm{fmol/s}$	Calculated from pituitary T_3-concentration (22)
K_{M2}	Dissociation constant of 5'-deiodinase type II	$1\,\mathrm{nmol/l}$	(23)

Symbol	Description	Value	Origin
α_{32}	Dilution factor for central T_3	$1.3 \cdot 10^5\ \mathrm{l}^{-1}$	Calculated from volume of distribution 7.6 µl (2)
β_{32}	Clearance exponent for central T_3	$8.3 \cdot 10^{-4}\ \mathrm{s}^{-1}$	Calculated from intra-cellular half-life of 15 min (24, 25)
α_{S2}	Dilution factor for pituitary TSH	$2.6 \cdot 10^5\ \mathrm{l}^{-1}$	Calculated from volume of distribution (2) of $3.8\ \mu\mathrm{l}$
β_{S2}	Clearance exponent for pituitary TSH	$140\ \mathrm{s}^{-1}$	Estimated, corresponding to half-life of $5\ \mathrm{ms}$ (2)
D_R	Damping constant for central T_3	$100\,\mathrm{pmol/l}$	(26)
G_R	Maximum gain of $TR\beta$-receptors	$1\,\mathrm{mol/s}$	Value unknown, normalized to 1 (magnitude of feedback is determined by L_S) (2)
S_S	Brake constant of ultrashort feedback	$100\,\mathrm{l/mIU}$	Determined according to values from (27)
D_S	Damping constant for TSH inside the pituitary	$50\,\mathrm{mIU/l}$	Determined according to values from (27)
K_{30}	Dissociation constant T_3-TBG	$2 \cdot 10^9\ \mathrm{l/mol}$	(18)
K_{31}	Dissociation constant T_3-IBS	$2 \cdot 10^9\ \mathrm{l/mol}$	Value unknown, adapted to extra-cellular dissociation constant (2)
K_{41}	Dissociation constant T_4-TBG	$2 \cdot 10^{10}\ \mathrm{l/mol}$	(18)
K_{42}	Dissociation constant T_4-$TBPA$	$2 \cdot 10^8\ \mathrm{l/mol}$	(18)
α_{th}	Dilution factor for $T_{4,th}$	$250\,\mathrm{l}^{-1}$	Based on an assumed volume of distribution of $4\ \mathrm{ml}$
β_{th}	Clearance Exponent for $T_{4,th}$	$4.4 \cdot 10^{-6}\ \mathrm{s}^{-1}$	Calculated from plasma half-life of 44 h (28)
k_{Dio}	Stimulation constant of thyroidal D1 and D2	$1\ \mathrm{mIU/l}$	(3)
K_{MCT8}	Michaelis-Menten constant MCT8	$4.7 \cdot 10^{-6}\ \mathrm{mol/l}$	(29)
m_{D3}	Oral dose of L-T_3	compare Section 3 of the main article	Determined by means of the MPC
m_{D4}	Oral dose of L-T_4	compare Section 3 of the main article	Determined by means of the MPC
t_l	Time interval between two medication intakes	8, 12 or 24h	Depends on the number of daily intakes

Symbol	Description	Value	Origin
k_{13}	Dissolution rate constant for L-T_3	$3.7825\,\mathrm{h}^{-1}$	Fitted to real measurements of (9)
k_{14}	Dissolution rate constant for L-T_4	$9.8379\,\mathrm{h}^{-1}$	Fitted to real measurements of (9)
k_{23}	Direct gut excretion rate constant for L-T_3	$0.8246\,\mathrm{h}^{-1}$	Fitted to real measurements of (9)
k_{24}	Direct gut excretion rate constant L-T_4	$3.5187\,\mathrm{h}^{-1}$	Fitted to real measurements of (9)
k_{33}	Absorption rate into the plasma constant for L-T_3	$2.9580\,\mathrm{h}^{-1}$	Fitted to real measurements of (9)
k_{34}	Absorption rate into the plasma constant for L-T_4	$6.3931\,\mathrm{h}^{-1}$	Fitted to real measurements of (9)
G_{D1}	Maximum activity of 5'deiodinase type I	$1.98 \cdot 10^{-8}\,\mathrm{mol/s}$	Fitted to real measurements of (2)
G_{T3}	Maximum activity of direct T_3 synthesis	$20.91 \cdot 10^{-14}\,\mathrm{mol/s}$	Fitted to real measurements of (2)
G_{MCT8}	Maximum activity of the MCT8	$1.94 \cdot 10^{-6}\,\mathrm{mol/s}$	Fitted to real measurements of (2)

REFERENCES

1 .Wolff TM, Veil C, Dietrich JW, Müller MA. Mathematical Modeling of Thyroid Homeostasis: Implications for the Allan-Herndon-Dudley Syndrome. *bioRxiv* (2022). doi:10.1101/2022.01.24.476744.

2 .Dietrich JW. *Der Hypophysen-Schilddrüsen-Regelkreis: Entwicklung und klinische Anwendung eines nichtlinearen Modells*. Ph.D. thesis (2001). doi:10.13140/RG.2.1.4845.9368.

3 .Berberich J, Dietrich JW, Hoermann R, Müller MA. Mathematical modeling of the pituitary-thyroid feedback loop: Role of a TSH-T3-Shunt and Sensitivity Analysis. *Frontiers in endocrinology* **9** (2018) article 91. doi:10.3389/fendo.2018.00091.

4 .Mak PH, DiStefano JJ. Optimal control policies for the prescription of thyroid hormones. *Mathematical Biosciences* **42** (1978) 159–186. doi:10.1016/0025-5564(78)90094-9.

5 .Rawlings JB, Mayne DQ, Diehl M. *Model predictive control: theory, computation, and design* (Nob Hill Publishing Madison, WI), 2 edn. (2020).

6 .Pilz S, Theiler-Schwetz V, Malle O, Steinberger E, Trummer C. Hypothyreose: Guidelines, neue erkenntnisse und klinische praxis. *Journal für Klinische Endokrinologie und Stoffwechsel* **13** (2020) 88–95. doi:10.1007/s41969-020-00114-9.

7 .Andersson JAE, Gillis J, Horn G, Rawlings JB, Diehl M. CasADi – A software framework for nonlinear optimization and optimal control. *Mathematical Programming Computation* **11** (2019) 1–36. doi:10.1007/s12532-018-0139-4.

8 .Jonklaas J, Davidson B, Bhagat S, Soldin SJ. Triiodothyronine levels in athyreotic individuals during levothyroxine therapy. *Jama* **299** (2008) 769–777.

9 .Hasselström K, Siersbæk-Nielsen K, Faber J, Kirkegaard C, Friis T, et al. The bioavailability of thyroxine and 3, 5, 3'-triiodothyronine in normal subjects and in hyper-and hypothyroid patients. *European Journal of Endocrinology* **110** (1985) 483–486.

10 .DiStefano J, Jonklaas J. Predicting optimal combination LT4 + LT3 therapy for hypothyroidism based on residual thyroid function. *Frontiers in endocrinology* **10** (2019) 746–746. doi:10.3389/fendo.2019.00746.

11 .Neubert D. Schilddrüsenhormon. *Endokrinologie II* **19** (1977) 65–212.

12 .M T Hays JT MR Broome. A multicompartmental model for iodide, thyroxine, and triiodothyronine metabolism in normal and spontaneously hyperthyroid cats*. *Endocrinology* **122** (1988) 2444–2461. doi:10.1210/endo-122-6-2444.

13 .Rondeel JMM, De Greef WJ, Van Der Schoot P, Karels B, Klootwijk W, Visser TJ. Effect of thyroid status and paraventricular area lesions on the release of thyrotropin-releasing hormone and catecholamines into hypophysial portal blood. *Endocrinology* **123** (1988) 523–527. doi:10.1210/endo-123-1-523.

14 .D'angelo S, Paul D, Wall N, Lombardi D. Pituitary thyrotropin (TSH) rebound phenomenon and kinetics of secretion in the goitrous rat: differential effects of thyroxine on synthesis and release of TSH. *Endocrinology* **99** (1976) 935–943.

15 .Okuno A, Taguchi T, Nakayama K, Takimoto M. Kinetic analysis of plasma TSH dynamics after TRH stimulation. *Hormone and Metabolic Research* **11** (1979) 293–295.

16 .Le Dafniet M, Brandi AM, Kujas M, Chanson P, Peillon F. Thyrotropin-releasing hormone (TRH) binding sites and thyrotropin response to TRH are regulated by thyroid hormones in human thyrotropic adenomas. *European journal of endocrinology* **130** (1994) 559–564.

17 .Odell WD, Utiger RD, Wilber JF, Condliffe PG. Estimation of the secretion rate of thyrotropin in man. *J Clin Invest* **46** (1967) 953–959. doi:10.1172/JCI105601.

18 .Li G, Liu B, Liu Y. A dynamical model of the pulsatile secretion of the hypothalamo-pituitary-thyroid axis. *Bio Systems* **35** (1995) 83–92.

19 .Dumont JE, Vassart G. Thyroid regulation. *Endocrinology DeGroot, WB Saunders* (1995).

20 .Greenspan F. Basic & clinical endocrinology, chapter the thyroid gland. *Stamford, CT, Appleton & Lange* (1997) 192–262.

21 .M G. *Metabolismus der Schilddrüsenhormone 1* (, Stuttgart, New York,: Thieme) (1988).

22 .Doorn JV, Roelfsema F, Heide DVD. Concentrations of thyroxine and 3, 5, 3-triiodothyronine at 34 different sites in euthyroid rats as determined by an isotopic equilibrium technique. *Endocrinology* **117** (1985) 1201–1208.

23 .Visser TJ, Kaplan MM, Leonard JL, Larsen PR. Evidence for two pathways of iodothyronine 5-deiodination in rat pituitary that differ in kinetics, propylthiouracil sensitivity, and response to hypothyroidism. *J Clin Invest* **71** (1983) 992–1002. doi:10.1172/JCI110854.

24 .Oppenheimer JH, Bernstein G, Hasen J. Estimation of rapidly exchangeable cellular thyroxine from the plasma disappearance curves of simultaneously administered thyroxine-131i and albumin-125i. *J Clin Invest* **46** (1967) 762–777. doi:10.1172/JCI105577.

25 .Benvenga S, Robbins J. Thyroid hormone efflux from monolayer cultures of human fibroblasts and hepatocytes. Effect of lipoproteins and other thyroxine transport proteins. *Endocrinology* **139** (1998) 4311–4318. doi:10.1210/endo.139.10.6231.

26 .Lazar MA, Chin WW, et al. Nuclear thyroid hormone receptors. *The Journal of clinical investigation* **86** (1990) 1777–1782.

27 .Kakita T, Laborde N, Odell WD. Autoregulatory control of thyrotropin in rabbits. *Endocrinology* **114** (1984) 2301–2305. doi:10.1210/endo-114-6-2301.

28 .Di Cosmo C, Liao XH, Dumitrescu AM, Philp NJ, Weiss RE, Refetoff S. Mice deficient in MCT8 reveal a mechanism regulating thyroid hormone secretion. *The Journal of clinical investigation* **120** (2010) 3377–88.

29 .Friesema ECH, Ganguly S, Abdalla A, Manning Fox JE, Halestrap AP, Visser TJ. Identification of monocarboxylate transporter 8 as a specific thyroid hormone transporter. *The Journal of biological chemistry* **278** (2003) 40128–35.
